# Comparative genomic analysis provides insights into the genetic diversity and pathogenicity of the genus *Brucella*

**DOI:** 10.3389/fmicb.2024.1389859

**Published:** 2024-04-24

**Authors:** Zilong Yang, Zili Chai, Xia Wang, Zehan Zhang, Fengwei Zhang, Fuqiang Kang, Wenting Liu, Hongguang Ren, Yuan Jin, Junjie Yue

**Affiliations:** Laboratory of Advanced Biotechnology and State Key Laboratory of Pathogen and Biosecurity, Beijing Institute of Biotechnology, Beijing, China; ^2^Department of Laboratory Medical Center, General Hospital of Northern Theater Command, Shenyang, China

**Keywords:** *Brucella*, pangenome, horizontal gene transfer, mobile genetic elements, virulence factors, antibiotic resistance genes

## Abstract

Some *Brucella* spp. are important pathogens. According to the latest prokaryotic taxonomy, the *Brucella* genus consists of facultative intracellular parasitic *Brucella* species and extracellular opportunistic or environmental *Brucella* species. Intracellular *Brucella* species include classical and nonclassical types, with different species generally exhibiting host preferences. Some classical intracellular *Brucella* species can cause zoonotic brucellosis, including *B. melitensis*, *B. abortus*, *B. suis*, and *B. canis*. Extracellular *Brucella* species comprise opportunistic or environmental species which belonged formerly to the genus *Ochrobactrum* and thus nowadays renamed as for example *Brucella intermedia* or *Brucella anthropi*, which are the most frequent opportunistic human pathogens within the recently expanded genus *Brucella*. The cause of the diverse phenotypic characteristics of different *Brucella* species is still unclear. To further investigate the genetic evolutionary characteristics of the *Brucella* genus and elucidate the relationship between its genomic composition and prediction of phenotypic traits, we collected the genomic data of *Brucella* from the NCBI Genome database and conducted a comparative genomics study. We found that classical and nonclassical intracellular *Brucella* species and extracellular *Brucella* species exhibited differences in phylogenetic relationships, horizontal gene transfer and distribution patterns of mobile genetic elements, virulence factor genes, and antibiotic resistance genes, showing the close relationship between the genetic variations and prediction of phenotypic traits of different *Brucella* species. Furthermore, we found significant differences in horizontal gene transfer and the distribution patterns of mobile genetic elements, virulence factor genes, and antibiotic resistance genes between the two chromosomes of *Brucella*, indicating that the two chromosomes had distinct dynamics and plasticity and played different roles in the survival and evolution of *Brucella*. These findings provide new directions for exploring the genetic evolutionary characteristics of the *Brucella* genus and could offer new clues to elucidate the factors influencing the phenotypic diversity of the *Brucella* genus.

## 1 Introduction

*Brucella* is a Gram-negative, facultative intracellular parasitic bacteria. *Brucella* can infect humans and a variety of animals, causing brucellosis (Cloeckaert et al., 2020). Different *Brucella* species have unique host preferences, virulence, and pathogenicity (Rajendhran, 2021). *B. abortus*, *B. melitensis*, *B. suis*, and *B. canis* can be transmitted between humans and animals (Marzetti et al., 2013). *B. melitensis* is the most common zoonotic *Brucella* species, followed by *B. abortus*, *B. suis*, and *B. canis* (Cloeckaert et al., 2024). Recent studies have indicated that *B. neotomae* and *B. inopinata* may also have the potential for zoonotic transmission, suggesting a potentially wider distribution of zoonotic potential within the *Brucella* genus (Cloeckaert et al., 2024; Vergnaud et al., 2024). Brucellosis poses significant risks to human health (Franco et al., 2007). Massive outbreaks of brucellosis among livestock can result in significant economic losses in the livestock industry (Hao et al., 2023). Additionally, due to the pathogenicity, high transmissibility, and aerosol transmission of *Brucella*, there is a potential risk of *Brucella* being used for the development of bioterrorism agents (Doganay and Doganay, 2013). Genomic analysis revealed a highly conserved genome structure among different *Brucella* species. The genomic similarity within the *Brucella* genus is above 97%, and the GC content is approximately 57%. The average genome size of *Brucella* isolates is 3.3 Mb, consisting of two circular chromosomes of different sizes, approximately 2.1 Mb and 1.2 Mb, respectively (Rajendhran, 2021).

*Ochrobactrum* are Gram-negative, plant-associated soil bacteria (Lebuhn et al., 2000; Kettaneh et al., 2003). *O. anthropi*, *O. intermedium*, and *O. pseudintermedium* are opportunistic pathogens that can occasionally infect immunocompromised humans (Leclercq et al., 2019). The *Ochrobactrum* genome spans from 4.7 Mb to 8.3 Mb (Teyssier et al., 2005). Most *Ochrobactrum* genomes contain two circular chromosomes and multiple plasmids (Teyssier et al., 2005).

The phylogenetic relationships between *Brucella* and *Ochrobactrum* are extremely close. The phylogenetic analysis of *Brucella* and *Ochrobactrum* by Leclercq et al. indicated that the genus *Ochrobactrum* was divided into two branches, with one branch mainly composed of environmental species, while the other branch encompassed the entire genus *Brucella* and opportunistic pathogens (primarily *O. anthropi*, *O. intermedium*, and *O. pseudintermedium*). All *Brucella* species formed a single genomic species within the *Ochrobactrum* branch (Leclercq et al., 2019). Recently, Hördt et al. proposed that the *Ochrobactrum* genus and the *Brucella* genus should be merged into a single genus for the following two reasons. First, phylogenetic analysis based on 16S rRNA indicated that the entire *Brucella* genus was nested within the *Ochrobactrum* genus. Second, despite differences in virulence between *Brucella* and *Ochrobactrum*, other genera, such as *Mycobacterium*, *Burkholderia*, and *Yersinia*, also contain populations with different risk levels (Hordt et al., 2020).

Based on the aforementioned whole-genome phylogenetic analysis results and related proposals, the genera *Brucella* and *Ochrobactrum* have been merged into a single genus, *Brucella* (Centers for Disease Control and Prevention, 2022; DSMZ, 2024). The *Brucella* genus is currently comprised of two major categories: the facultative intracellular parasitic *Brucella* (formerly known as the *Brucella* genus) and the extracellular *Brucella* (formerly known as the *Ochrobactrum* genus) (Aljanazreh et al., 2023). The intracellular *Brucella* are further divided into classical intracellular *Brucella* and nonclassical intracellular *Brucella*. Classical intracellular *Brucella* consists of the six initially identified species and newer species such as *B. ceti*, *B. pinnipedialis*, *B. microti*, and *B. papionis*. Nonclassical intracellular *Brucella*, more distantly related phylogenetically to classic species, includes isolates from red foxes, Australian rodents, several frog species, rays, and human cases. These nonclassical intracellular *Brucella* represent several potential new species, with *B. inopinata* isolated from human cases being the sole representative (Cloeckaert et al., 2020).

In this study, we conducted a comparative genomic analysis of the latest *Brucella* genus utilizing publicly available genome data from the NCBI Genome database. The main objective of this study was to investigate the genetic evolutionary characteristics of the latest *Brucella* genus and elucidate the relationship between the genomic composition and prediction of phenotypic traits of *Brucella*.

## 2 Materials and methods

### 2.1 Genome collection

With time more *Brucella* genomes are constantly becoming available. In this study, *Brucella* genomes with “complete” and “chromosome” assembly levels were collected from the Genome database of NCBI (accessed on 11 November 2022). After quality control was performed using CheckM 1.2.1 (Parks et al., 2015), 255 genomes with completeness greater than 90% were retained for subsequent analysis. The sample information of the 255 genomes was collected (Supplementary Figure S1; Supplementary Table S1). These genomes were obtained from 244 strains of 14 *Brucella* species and 11 strains that have not yet been identified. Moreover, these strains were collected from 2001 to 2022 in multiple countries, including Italy, China, Norway, and others. Additionally, these *Brucella* strains were isolated from a wide range of animal hosts and environments, such as humans, water buffaloes, cattle, pigs, dogs, sheep, and so on. The genomes of *B. sp.* 09RB8471 (GCA_001971625.1) and *B. melitensis* 6144 (GCA_023796775.1) were separately compared for collinearity with the genome of *B. melitensis* 16M (GCA_000007125.1) using Mauve 20150226 (Darling et al., 2004), and chromosome numbering errors in these two genomes were corrected (Supplementary Figure S2).

### 2.2 Gene annotation

To ensure comparability, 255 *Brucella* genomes were annotated using Prokka 1.14.6 (Seemann, 2014). EggNOG-mapper 2.1.5 was utilized for functional annotation and for providing COG (clusters of orthologous groups) information for all the genes (Cantalapiedra et al., 2021). Virulence factor genes (VFGs) and antibiotic resistance genes (ARGs) in the *Brucella* genome were identified using the Virulence Factors of Pathogenic Bacteria Database (VFDB) (accessed on 27 March 2023) (Liu et al., 2022) and the Comprehensive Antibiotic Resistance Database (CARD) (version: card-data.3.2.9) (Alcock et al., 2023), respectively.

### 2.3 Pangenome analysis

Gene families in the pangenome of 255 *Brucella* strains were identified using OrthoFinder 2.5.4 (-S diamond-M msa-T fasttree) (Emms and Kelly, 2015; Emms and Kelly, 2019). Core gene families (distributed in all 255 genomes), accessory gene families (distributed in at least 2 genomes, at most 254 genomes), and strain-specific genes (distributed only in one genome) were identified based on the OrthoFinder results. The pangenome growth curve was modeled using Heaps’ law (y = Ax^B^ + C) (Heaps, 1978; Tettelin et al., 2008), where y represents the number of gene families and x represents the number of genomes. Additionally, a 0–1 matrix file representing the presence/absence pattern of gene families was generated based on the results of OrthoFinder. To obtain a pan tree file, clustering analysis was conducted on the 0–1 matrix file using the R package “pheatmap” (clustering_method = “ward. D2,” clustering_distance = “manhattan”). Moreover, to compare the core tree and pan tree topology, the normalized matching-cluster (nMC) and normalized Robinson–Foulds (nRF) scores were calculated using TreeCmp 2.0-b76 (Bogdanowicz et al., 2012). These two scores range from 0 to 1, with higher values indicating greater differences in the topology between the two trees. Finally, a tanglegram of the two trees was generated using the R package “ape.”

### 2.4 Phylogenetic analysis

The average nucleotide identity (ANI) values of the pairwise comparison of 255 *Brucella* genomes were calculated using Pyani 0.2.12 (−m ANIm) (Pritchard et al., 2016). The digital DNA–DNA hybridization (dDDH) values between each of the 255 genomes and *B. melitensis* 16M were calculated using GGDC 3.0 (Meier-Kolthoff et al., 2013, 2022). The dDDH values of pairwise comparison of the 25 reference genomes from different *Brucella* species were also calculated using GGDC 3.0. A phylogenetic tree of the 255 *Brucella* strains was constructed based on the core single-copy orthologous genes. *Falsochrobactrum ovis* DSM 26720 (GCA_003259955.1) was selected as an outgroup. Specifically, all orthologous groups within the 255 *Brucella* genomes and one outgroup genome were identified using OrthoFinder 2.5.4. Then, all the core single-copy orthologous gene sequences were aligned using Muscle 3.8.1551 (Edgar, 2021). The alignment products were subsequently trimmed using the Trimal 1.4.rev15 (Capella-Gutierrez et al., 2009). Subsequently, AMAS.py (Borowiec, 2016) was used to concatenate all the alignment results. Finally, using the concatenated multiple sequence alignment file, a maximum likelihood (ML) tree was built using Iqtree 2.0.3 (−m MFP-B 1000 --bnni-T AUTO --seed 123) (Minh et al., 2020). The phylogenetic tree was visualized using ITOL (Letunic and Bork, 2021).

### 2.5 Identification of horizontal transfer gene families and mobile genetic elements

HGTector 2.0b3 (Zhu et al., 2014) was used to identify horizontal transfer gene families (HTGFs) in 255 *Brucella* genomes that have transferred from various organisms outside the family *Brucellaceae* to the genus *Brucella*. *Brucella* was utilized as the self-group, and *Brucellaceae* was utilized as the close group.

Insertion sequences (ISs) in 255 *Brucella* genomes were identified using Isfinder (Siguier et al., 2006). Prophages were detected using PHASTER (Arndt et al., 2016). Integrative conjugative elements (ICEs) and integrative mobilizable elements (IMEs) were identified using ICEfinder (Liu et al., 2019).

## 3 Results and discussion

### 3.1 The pangenome of the *Brucella* genus is dynamically open, and the two chromosomes exhibit different levels of openness

**Figure 1:**
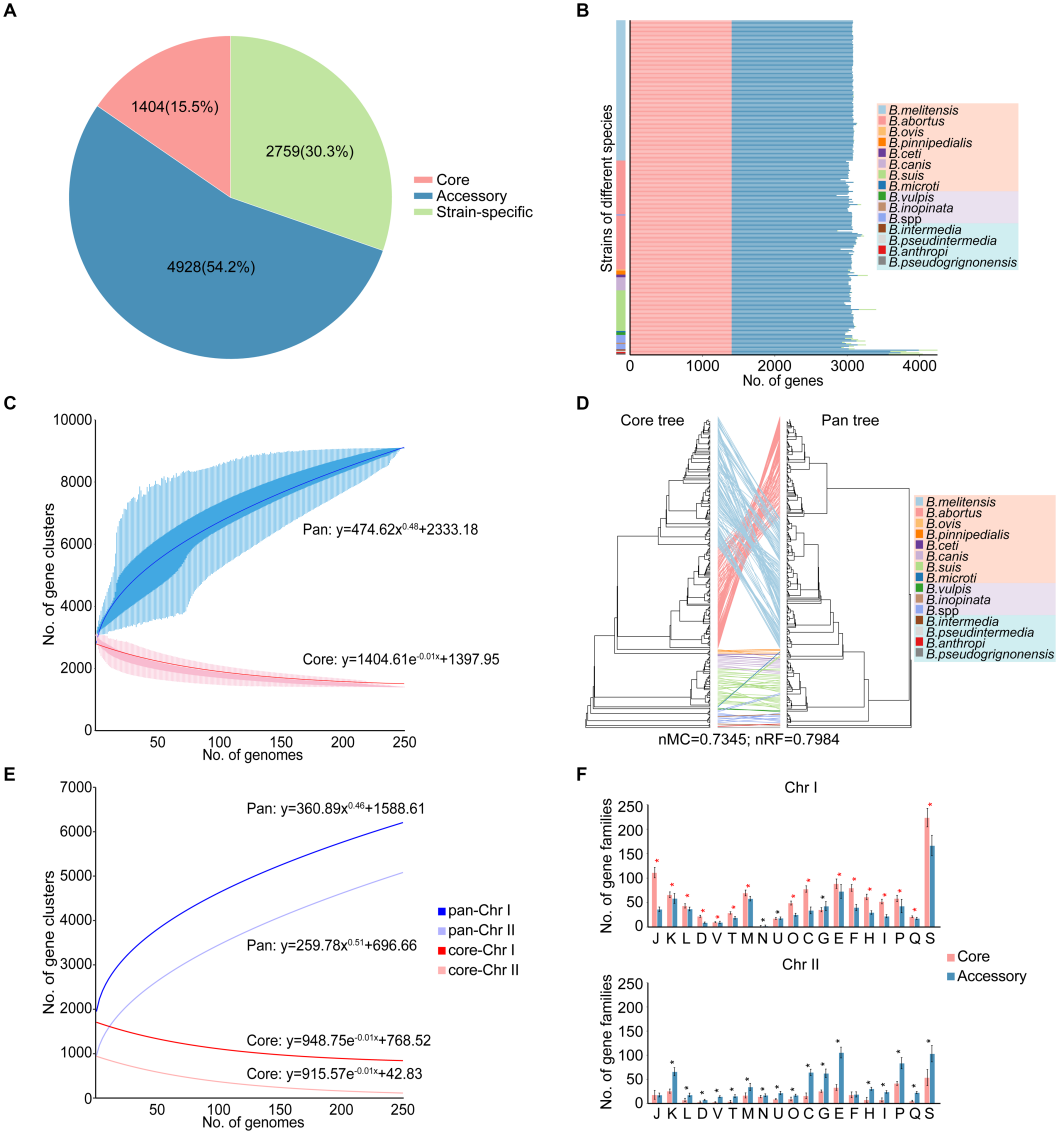
Characteristics of the pangenome of the *Brucella* genus. **(A)** The pangenome composition. **(B)** The numbers of core gene families, accessory gene families and strain-specific genes in each strain. **(C)** The accumulation curves of the pangenome and core genome of the *Brucella* genus. The blue curve shows an open pangenome; the deduced pangenome size was y = 474.62x^0.48^ + 2333.18. The red curve shows the core genome, and the deduced core genome size was y = 1404.61e^–0.01x^ + 1397.95. **(D)** The tanglegram shows the differences in the topological structures between the core tree and the pan tree. A core tree was created based on core single-copy orthologues. The pan tree was created based on a binary panmatrix (gene presence/absence matrix). **(E)** The accumulation curves of the pan-Chr I and pan-Chr II of the *Brucella* genus. The dark color represents Chr I, while the light color represents Chr II. **(F)** The distribution and COG functional characteristics of the core and accessory gene families on Chr I and Chr II. * *t*–test *p* < 0.05.

Xiaowen Yang et al. and Jagadesan Sankarasubramanian et al. separately conducted studies on the pangenomes of eight (Yang et al., 2016) and 11 (Sankarasubramanian et al., 2017) intracellular *Brucella* species, respectively. The 255 strains in our study included not only 10 species of intracellular *Brucella* but also four species of extracellular *Brucella*. Therefore, compared to those in the aforementioned two studies, the pangenome in our study more comprehensively reflected the species diversity and genetic polymorphisms of the *Brucella* genus. A total of 9,091 gene families were identified in the pangenome analysis, including 1,404 core gene families, 4,928 accessory gene families, and 2,759 strain-specific genes (Figure 1A; Supplementary Table S2). Several *Brucella* strains harbored more accessory gene families or strain-specific genes than did the other strains, resulting in a high proportion of accessory gene families and strain-specific genes in the pangenome (Figure 1B). In particular, *B. intermedia*, *B. pseudintermedia*, *B. anthropi*, and *B. pseudogrignonensis* harbored more accessory gene families and strain-specific genes. With the inclusion of additional genomes, the pangenome expanded continuously (Figure 1C), following Heaps’ law (y = Ax^B^ + C). Notably, the parameter B was greater than 0; thus, the pangenome in this study was open, suggesting that a large number of genomes need to be analyzed to fully characterize the complete gene pool of the *Brucella* genus. The open pangenome reflected the genetic diversity of the *Brucella* genus, indicating the possibility of genetic exchange among the gene pool of *Brucella* and the external environment. In contrast, the core genome exhibited the opposite trend (Figure 1C), as depicted by a fitted model (y = Ae^Bx^ + C), in which the size gradually decreased with the addition of new genomes.

To elucidate the impact of core gene families, accessory gene families, and strain-specific genes on the phylogeny of the *Brucella* genus, a core tree and a pan tree were constructed and compared (Figure 1D). The nMC and nRF scores were computed to assess the differences in tree topology between the core tree and the pan tree. In this study, the nMC and nRF scores were 0.7345 and 0.7984, respectively, indicating substantial disparities between the core tree and pan tree of the *Brucella* genus. The presence of a large number of accessory gene families and strain-specific genes in the open pangenome of the *Brucella* genus led to significant differences between the core tree and the pan tree. Furthermore, on the core tree and pan tree, extracellular *Brucella* and intracellular *Brucella* were significantly separated. Classical intracellular *Brucella* and nonclassical intracellular *Brucella* were separated from each other on the core tree, but there was a crossover phenomenon on the pan tree. This indicates a certain difference between extracellular *Brucella* and intracellular *Brucella* in core-pangenome assemblies.

The genomes of *Brucella* species are composed of two chromosomes. Pan-chromosome analyses were conducted on different chromosomes of *Brucella*. Based on Heaps’ law (y = Ax^B^ + C), the openness of chromosome II (Chr II) was greater than that of chromosome I (Chr I) (B_Chr II_ > B_Chr I_) (Figure 1E), suggesting that Chr II may be more dynamic than Chr I. The two chromosomes of *Brucella* are considered to have different evolutionary origins, and the smaller Chr II could be derived from a mega-plasmid possessing many dispensable genes (Bavishi et al., 2010). The functional and distribution characteristics of the core and accessory gene families on Chr I and Chr II were further explored, respectively (Figure 1F). Many core and accessory gene families were distributed on Chr I, with the core gene families being dominant, indicating that Chr I of *Brucella* was relatively conserved and stable. Except for the core gene families associated with “N: cell motility,” “U: intracellular trafficking, secretion, and vesicular transport,” and “G: carbohydrate transport and metabolism,” the abundance of core gene families related to other functions was significantly higher than that of accessory gene families on Chr I (*t*-test, *p* < 0.05). In contrast, the accessory gene families were dominant on Chr II, indicating that Chr II of *Brucella* was more dynamic and plastic and served as the primary reservoir for genetic diversity in the *Brucella* genus. Except for the accessory gene families associated with “J: translation, ribosomal structure and biogenesis” and “F: nucleotide transport and metabolism,” the abundance of accessory gene families related to other functions was significantly higher than that of core gene families on Chr II (*t*-test, *p* < 0.05).

### 3.2 The classical intracellular *Brucella*, nonclassical intracellular *Brucella* and extracellular *Brucella* strains exhibited varying degrees of genomic heterogeneity

**Figure 2:**
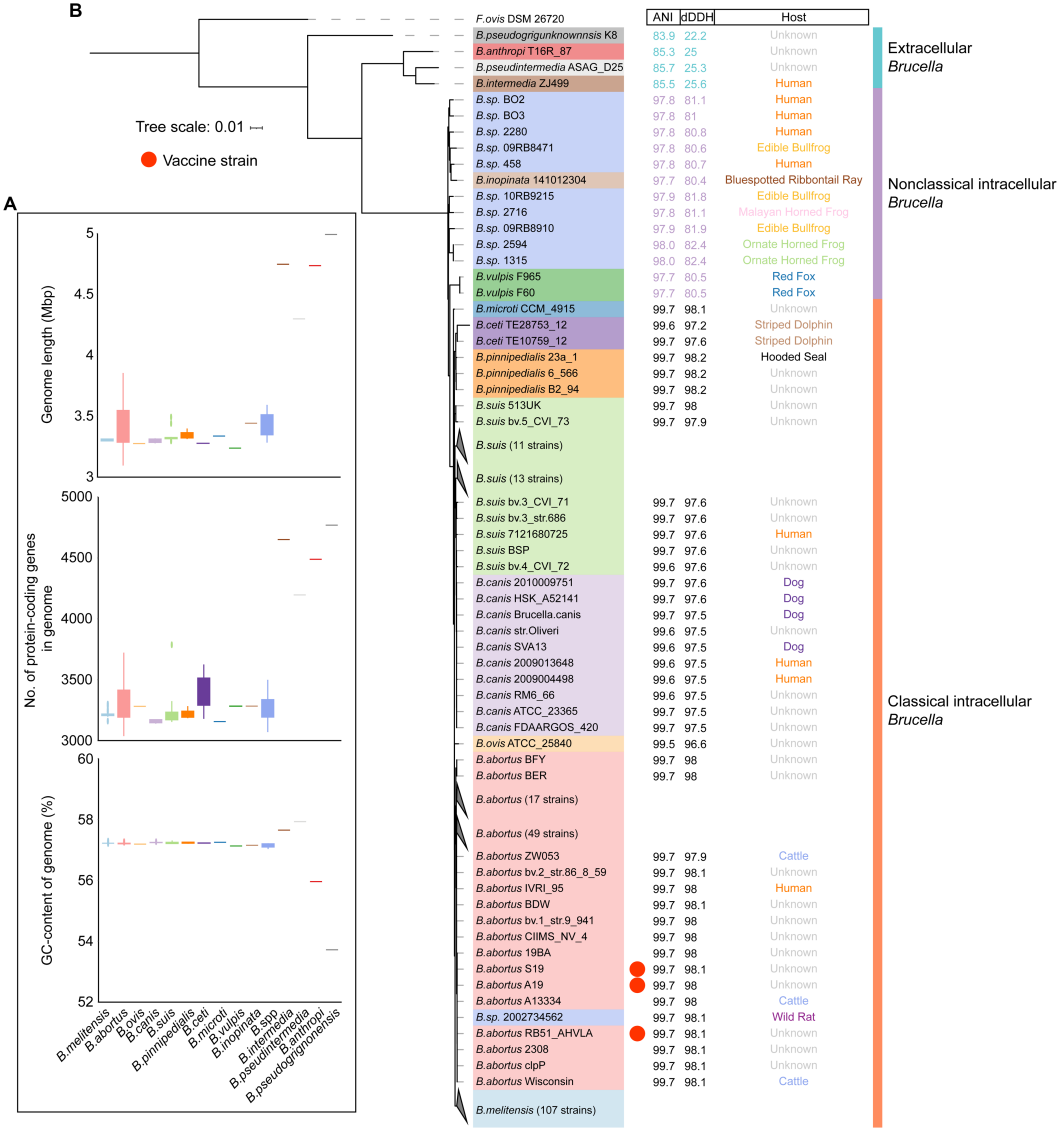
The genomic features and phylogenetic relationships of different *Brucella* species. **(A)** The genome length, number of protein-coding genes, and GC content of different *Brucella* species. **(B)** Phylogenomic tree showing the relationships between different *Brucella* species. The tree was created based on core single-copy orthologues using the maximum likelihood method in IQTREE and visualized using ITOL. *Falsochrobactrum ovis* DSM 26720 was used as an outgroup to root the tree. The strains from different *Brucella* species are differentiated using different colors. The ANI and dDDH values, and the host of each strain are displayed on the three columns.

To further explore the genetic diversity of the *Brucella* genus, a comparison was conducted on the genome sizes and GC contents of different *Brucella* species. It was evident that, with an average size of 4.69 Mb and an average of 4,327 protein-coding genes per genome, the genomes of extracellular *Brucella* species, such as *B. pseudogrignonensis*, *B. anthropi*, *B. pseudintermedia*, and *B. intermedia*, were significantly larger than those of facultative intracellular *Brucella* species (Figure 2A; Supplementary Table S1). The average genome size of facultative intracellular *Brucella* was 3.36 Mb, with an average of 2,971 protein-coding genes per genome. Furthermore, the genomic GC content of extracellular *Brucella* was significantly different from that of facultative intracellular *Brucella* (Figure 2A, Supplementary Table S1). The genomic GC content of facultative intracellular *Brucella* was highly conserved, ranging from 57.1 to 57.3%. However, different extracellular *Brucella* species had significant variations in genomic GC content, with the highest being 57.96% (*B. pseudintermedia* strain ASAG-D25) and the lowest being 53.71% (*B. pseudogrignonensis* strain K8). In summary, there were significant differences in genome size and GC content between facultative intracellular *Brucella* and extracellular *Brucella*. The genomes of different facultative intracellular *Brucella* species were conservative, while greater heterogeneity was observed among the genomes of different extracellular *Brucella* species.

To further assess the phylogenetic relationships among the different *Brucella* species, the core genome phylogenetic tree was constructed for 255 *Brucella* strains (Figure 2B). Within the extracellular *Brucella*, a separate branch was formed by *B. pseudogrignonensis*. *B. intermedia*, *B. pseudintermedia*, and *B. anthropi* clustered together. All the intracellular *Brucella* species clustered together. These results are consistent with the findings of Leclercq et al. They found that the entire *Brucella* genus was comprised of two major branches. One branch encompassed extracellular *Brucella* not involved in opportunistic infections, such as *B. pseudogrignonensis*. The other branch contained opportunistic pathogenic extracellular *Brucella* species (including *B. intermedia*, *B. pseudintermedia*, and *B. anthropi*) alongside intracellular *Brucella* capable of causing brucellosis (Leclercq et al., 2019). By calculating the ANI and dDDH values between each *Brucella* strain and the reference strain *B. melitensis* 16M, significant differences were identified among extracellular *Brucella*, nonclassical intracellular *Brucella*, and classical intracellular *Brucella* (Figure 2B). The ANI values between the extracellular *Brucella* strains and the reference strain *B. melitensis* 16M ranged from 83.9 to 85.7%. The ANI values between nonclassical intracellular *Brucella* strains and the reference strain *B. melitensis* 16M ranged from 97.7 to 98%. The ANI values between classical intracellular *Brucella* strains and the reference strain *B. melitensis* 16M were greater than 99%. The dDDH values exhibited the same characteristics as the ANI values (Figure 2B; Supplementary Figure S3). The dDDH values between extracellular *Brucella* strains and the reference strain *B. melitensis* 16M ranged from 22.2 to 25.3%. The dDDH values between nonclassical intracellular *Brucella* strains and the reference strain *B. melitensis* 16M ranged from 80.4 to 82.4%. The dDDH values between classical intracellular *Brucella* strains and the reference strain *B. melitensis* 16M were greater than 95%. ANI and dDDH values are widely used for prokaryotic taxonomic identification (Sentausa and Fournier, 2013). An ANI value greater than 96% or a DDH value greater than 70% was considered to indicate a single species (Konstantinidis and Tiedje, 2005; Goris et al., 2007). However, these two thresholds were not applicable for species identification within the *Brucella* genus. Nonetheless, the ANI and dDDH values still revealed significant genomic differences between extracellular *Brucella*, nonclassical intracellular *Brucella*, and classical intracellular *Brucella* (Supplementary Figure S4; Supplementary Figure S5).

Subsequently, the phylogenetic positions of all the undefined *Brucella* strains were examined. *B. sp.* 2002734562 clustered together with other *B. abortus* strains (Figure 2B), suggesting that *B. sp.* 2002734562 may be a member of *B. abortus*. The branch closest to *B. sp.* 2002734562 was occupied by *B. abortus* RB51-AHVLA (Supplementary Figure S3). *B. abortus* 2308, *B. abortus* S19, and *B. abortus* A19 were also close to the branch of *B. sp.* 2002734562. *B. abortus* RB51-AHVLA, *B. abortus* S19, and *B. abortus* A19 are vaccine strains of *B. abortus* (Refai, 2002; Moriyón et al., 2004; Wang et al., 2022). *B. abortus* 2308 is a virulent strain of *B. abortus* (Kellerman et al., 1970). Moreover, the other undefined strains of nonclassical *Brucella* exhibited a close phylogenetic relationship with *B. inopinata* (Figure 2B; Supplementary Figure S3). *B. sp.* 1315, *B. sp.* 2594, *B. sp.* 09RB8910, *B. sp.* 2716, and *B. sp.* 10RB9215 clustered together. Interestingly, these five strains were all isolated from frogs or toads. *B. sp.* 09RB8471 was also isolated from a frog and clustered together with *B. inopinata* 141012304 and 4 undefined strains isolated from humans. Although these 10 undefined strains and *B. inopinata* were separated into two clades, considering that the ANI and dDDH values of the members from the two clades were generally similar, we inferred that these undefined strains may come from the same species, such as *B. inopinata*.

### 3.3 Horizontal gene transfer was significantly different between the two chromosomes of *Brucella*, and each *Brucella* species had unique HGT patterns

**Figure 3:**
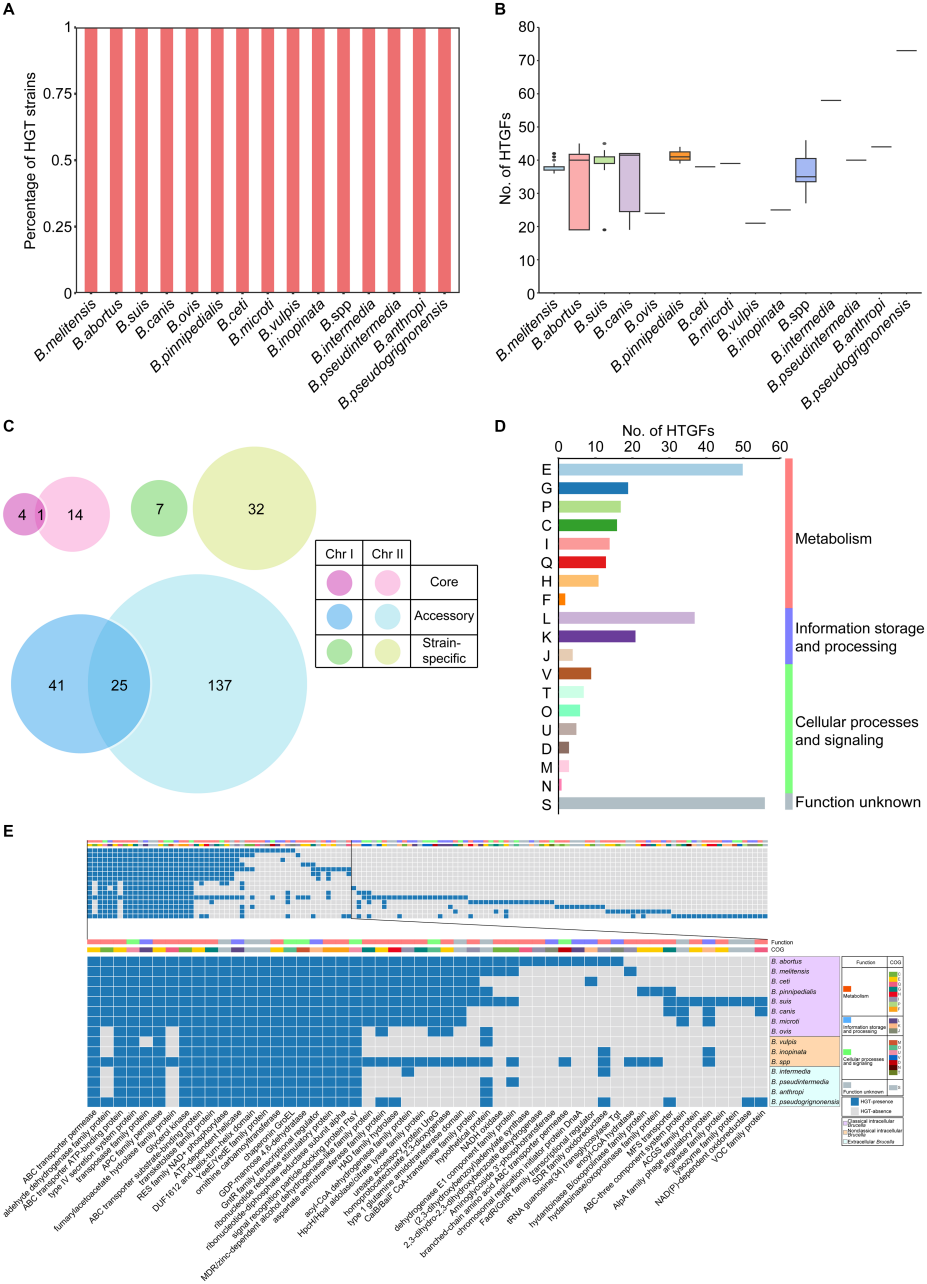
Characteristics of HGT within the *Brucella* genus. **(A)** The percentage of strains with HTGFs of different *Brucella* species. **(B)** The number of HTGFs carried by each genome of different *Brucella* species. **(C)** The distribution of HTGFs on two chromosomes and the pangenome of *Brucella*. **(D)** COG categories of HTGFs. **(E)** The products encoded by horizontal transfer genes of different *Brucella* species.

Horizontal gene transfer (HGT) refers to the transfer of genetic material between different organisms that are not related to the parent or offspring. HGT is an important mechanism in bacterial evolution that can promote the diversity and adaptability of bacterial populations (Jain et al., 1999; Ochman et al., 2000). In our study, the open pangenome of the *Brucella* genus contained a large number of accessory gene families and strain-specific genes, suggesting that HGT may have played an important role in the evolution of *Brucella*. Using HGTector, a total of 261 horizontal transfer gene families (HTGFs) in 255 *Brucella* genomes were identified, which had been transferred from various organisms outside the family *Brucellaceae* to the genus *Brucella* (Supplementary Table S3). All genomes of different *Brucella* species were found to contain HTGFs (Figure 3A). Variations were found in the number of HGTFs among different *Brucella* species (Figure 3B). The highest number of HTGFs was found in the genome of *B. pseudogrignonensis*, followed by *B. intermedia*, with 73 and 58 HTGFs, respectively. The genomes of *B. ovis*, *B. vulpis*, and *B. inopinata* contained fewer HTGFs, averaging 21–25 HTGFs each. The genomes of other *Brucella* species each had an average of 33–44 HTGFs.

Subsequently, the distribution of the 261 HTGFs in the genome and their relationships with the pangenome of the *Brucella* genus were further analyzed (Figure 3C). Among the 261 HTGFs, 52 (19.9%) were exclusively distributed on Chr I, 183 (70.1%) were exclusively distributed on Chr II, and the remaining 26 (10%) were distributed on both chromosomes. Furthermore, 19 (7.3%) were core gene families, 203 (77.8%) were accessory gene families, and the remaining 39 (14.9%) were strain-specific genes. The size of Chr II of the *Brucella* genus was significantly smaller than that of Chr I (Rajendhran, 2021). However, Chr II contained more HTGFs than Chr I, suggesting that Chr II of the *Brucella* genus might have experienced more HGT events than Chr I. HGT is an important mechanism for bacterial evolution and adaptation (De la Cruz and Davies, 2000). Using Alien_hunter, Anish Bavishi et al. found that the level of HGT on Chr II of *B. melitensis* 16M and *B. anthropi* ATCC 49188 was greater than that on Chr I (Bavishi et al., 2010). Our study examined HGT in the whole *Brucella* genus, aligning with Anish Bavishi et al.’s findings, and showing a higher HGT level in Chr II compared to Chr I in *Brucella*. Anish Bavishi et al. proposed a plasmid-mediated origin for Chr II as the preferred model explaining this bias of HGT (Bavishi et al., 2010). In this model, a resident plasmid would acquire essential gene clusters through intragenomic gene transfer from Chr I and HGT from other species, leading to the formation of Chr II. This accessory chromosome (Chr II) may then acquire new genes via various mechanisms like HGT. With Chr I housing crucial housekeeping genes, Chr II likely evolved as an adaptive reservoir under selection pressure to accommodate different ecological niches and nutritional challenges and played a vital role in *Brucella*’s evolution and adaptation.

To further elucidate the functional characteristics of these HTGFs, the COG information of these gene families was analyzed. These HTGFs were involved in diverse biological functions (Figure 3D). Nearly half (48%) of the HTGFs were associated with metabolism. Approximately one-fifth (21%) of the HTGFs were related to information storage and processing. Twelve percent of the HTGFs were related to cellular processes and signaling. However, the functions of the remaining 19% of the HTGFs are still unclear. In particular, the proportion of HTGFs associated with “E: amino acid transport and metabolism” was the highest (19%). The proportion of HTGFs associated with “L: replication, recombination, and repair” was the second highest (14%). *Brucella* are amino acid prototrophs that can grow with ammonium and contain many amino acid/peptide/polyamine uptake genes (Ronneau et al., 2016). Our research revealed that HGT played a significant role in the acquisition of essential biological functions such as amino acid transport and metabolism in the *Brucella* genus.

The functional characteristics of the HTGFs were further compared between different *Brucella* species. These 261 HTGFs were found to encode a total of 134 different proteins by a BLAST search using the NCBI nr database (Figure 3E; Supplementary Figure S6). The HTGFs associated with gene products such as ABC transporter, type IV secretion system proteins, APC family permease, and 15 other types were widely distributed throughout the *Brucella* genus (Figure 3E). The fact that different *Brucella* species shared these HTGFs suggested that relevant HGT events may have occurred in the ancient ancestor of the *Brucella* genus. The HTGFs associated with the transposase family proteins were distributed in all *Brucella* species except for *B. vulpis*. These HTGFs may represent historical traces of previous HGT in *Brucella*, and they may also provide a certain material basis for *Brucella* genomes to undergo additional HGT.

In classical intracellular *Brucella*, the HTGFs of *B. abortus* and *B. suis* encoded the most gene products, with 41 types (Figure 3E). The HTGFs of *B. ovis* encoded the fewest gene products, with 23 types. Different classical intracellular *Brucella* species commonly contained HTGFs associated with 22 gene products, such as ABC transporter permease, ABC transporter ATP-binding proteins, type IV secretion system proteins, and so on. Compared with other classical intracellular *Brucella* species, *B. ovis* lacked HTGFs associated with aldehyde dehydrogenase family proteins, fumarylacetoacetate hydrolase family proteins, and MDR/zinc-dependent alcohol dehydrogenase-like family proteins, and so on. Additionally, the abundance of HTGFs associated with ABC transporter permease, ABC transporter ATP-binding proteins in *B. ovis* was relatively lower than in other classical intracellular *Brucella*, and these ABC transport systems were primarily associated with amino acid transport (Supplementary Figure S6). ABC transporters play a significant role in the antibiotic resistance and virulence of bacteria (van Veen and Konings, 1998; Paulley et al., 2007). Jenner et al. reported that *B. abortus*, *B. melitensis*, *B. suis*, *B. canis*, and *B. ovis* all contain a large number of ABC transport systems and that *B. ovis* has relatively fewer ABC transport systems than the other four species, primarily lacking polyamine transporters. Considering that *B. ovis* does not cause zoonotic infections, Jenner et al. speculated that the absence of ABC transport systems in *B. ovis* may be related to the virulence of *Brucella* (Jenner et al., 2009). In our study, HTGFs associated with ABC transport systems were relatively less abundant in *B. ovis* compared to other classical intracellular *Brucella* species. The differential distribution of HTGFs associated with ABC transport systems and other functional proteins between *B. ovis* and other classical intracellular *Brucella* species may be linked to the significant differences in zoonotic potential and host preferences between them. Additionally, *B. suis* and *B. canis* have a close phylogenetic relationship, with *B. canis* often nested within the branch of *B. suis* on the phylogenetic tree (Fretin et al., 2008). However, significant differences were found in their HTGFs. The HTGFs associated with ACGS family proteins, arginase family proteins, and lysozyme family proteins were unique to *B. suis* (Figure 3E). These HTGFs were not found in *B. canis*.

In nonclassical intracellular *Brucella*, *B. vulpis* harbored HTGFs related to 20 gene products, among which those associated with DEAD/DEAH box helicase were unique to *B. vulpis* (Supplementary Figure S6). *B. inopinata* harbored HTGFs related to 24 gene products but did not contain any unique HTGFs. The unclassified *Brucella* strains harbored HTGFs related to 59 gene products, among which those associated with 14 gene products such as FAD-dependent oxidoreductase, GNAT family N-acetyltransferase, and helicase RepA family proteins were specific to these unclassified *Brucella* strains.

Different extracellular *Brucella* species had HTGFs that correspond to 34–54 gene products (Supplementary Figure S6). The HTGFs associated with 14 gene products such as the AraC family transcriptional regulator, and M20 aminoacylase family proteins were unique to *B. intermedia*. The HTGFs related to 10 gene products including ACR3 family arsenite efflux transporter, and LysE family translocator were specific to *B. pseudintermedia*. The HTGFs associated with 11 gene products such as arginine deiminase, and histidinol dehydrogenase were unique to *B. anthropi*. Additionally, HTGFs related to 19 gene products like amidohydrolase, and ferrochelatase were specific to *B. pseudogrignonensis*.

In summary, significant differences in the distribution of HTGFs were found among classical intracellular *Brucella*, nonclassical intracellular *Brucella*, and extracellular *Brucella*. In particular, extracellular *Brucella* was found to contain a greater number of unique HTGFs than intracellular *Brucella*. Intracellular *Brucella* has a host preference and an intracellular lifestyle, which to some extent hinders the occurrence of HGT (Rouli et al., 2015). On the contrary, the free distribution of extracellular *Brucella* in the environment was more conducive to genetic exchange with other organisms. These findings indicate that HGT enhanced the genetic diversity of *Brucella* and facilitated the acquisition of various important biological functions.

### 3.4 The *Brucella* genomes harbored various mobile genetic elements, and the two chromosomes exhibited differential distributions of mobile genetic elements

#### 3.4.1 IS elements

**Figure 4:**
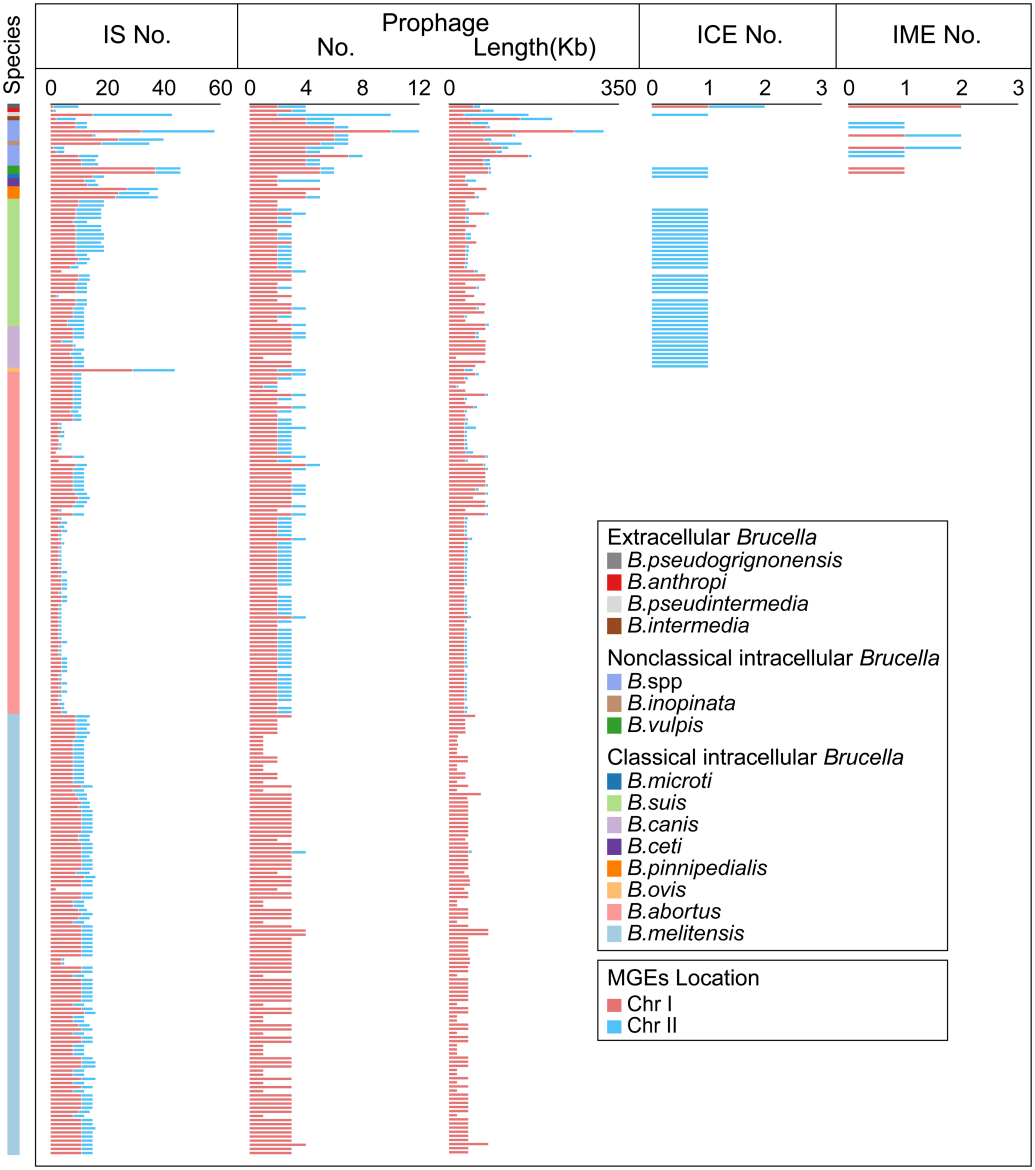
Distribution of MGEs in the *Brucella* genus. The presence of IS elements, prophages, ICEs, and IMEs, on Chr I and Chr II of each *Brucella* strain are shown.

Mobile genetic elements (MGEs) play a crucial role in HGT as important carriers for transmitting genetic information across cells, individuals, and species (Haudiquet et al., 2022). IS elements, one type of MGE, are small DNA fragments ranging in length from 700 bp to 2,500 bp (Aljanazreh et al., 2023). IS elements play a crucial role in promoting genomic plasticity and genetic variability. Beneficial mutations generated by IS transposition help bacteria overcome environmental challenges and adapt to new ecological niches (Vandecraen et al., 2017). In our study, IS elements were found in all 255 *Brucella* genomes (Figure 4; Supplementary Table S4). Each *Brucella* genome contained an average of 12 IS elements. Different *Brucella* species contained varying quantities of IS elements (Figure 4). The genomes of *B. ovis*, *B. pinnipedialis*, *B. vulpis*, *B. inopinata*, *B. pseudintermedia*, *B. sp.* 2280, and *B. sp.* 458 contained relatively high numbers of IS elements, with an average of 42. In particular, the genome of *B. sp.* 2280 had the highest number of IS elements, with as many as 58 IS elements. In contrast, the other *Brucella* genomes contained fewer IS elements, with an average of 11. IS elements were found on Chr I of all 255 *Brucella* genomes and were found on Chr II of 250 *Brucella* genomes (Figure 4). Furthermore, Significant differences were observed in the distribution of IS elements between intracellular and extracellular *Brucella*. Chr I of intracellular *Brucella* contained a greater number of IS elements than did Chr II. However, Chr I of extracellular *Brucella* contained a fewer number of IS elements than did Chr II. In particular, Chr I of *B. intermedia*, *B. pseudintermedia*, and *B. pseudogrignonensis* had fewer IS elements than did Chr II. Chr I and Chr II of *B. anthropi* contained an equal number of IS elements. The density of IS elements on the two chromosomes of classical intracellular *Brucella*, nonclassical intracellular *Brucella*, and extracellular *Brucella* varied significantly (Supplementary Figure S7). The average distribution density of IS elements on Chr I was significantly higher than that on Chr II in classical and nonclassical intracellular *Brucella*. Conversely, the average distribution density of insertion sequences on Chr I was significantly lower than that on Chr II in extracellular *Brucella*.

**Figure 5:**
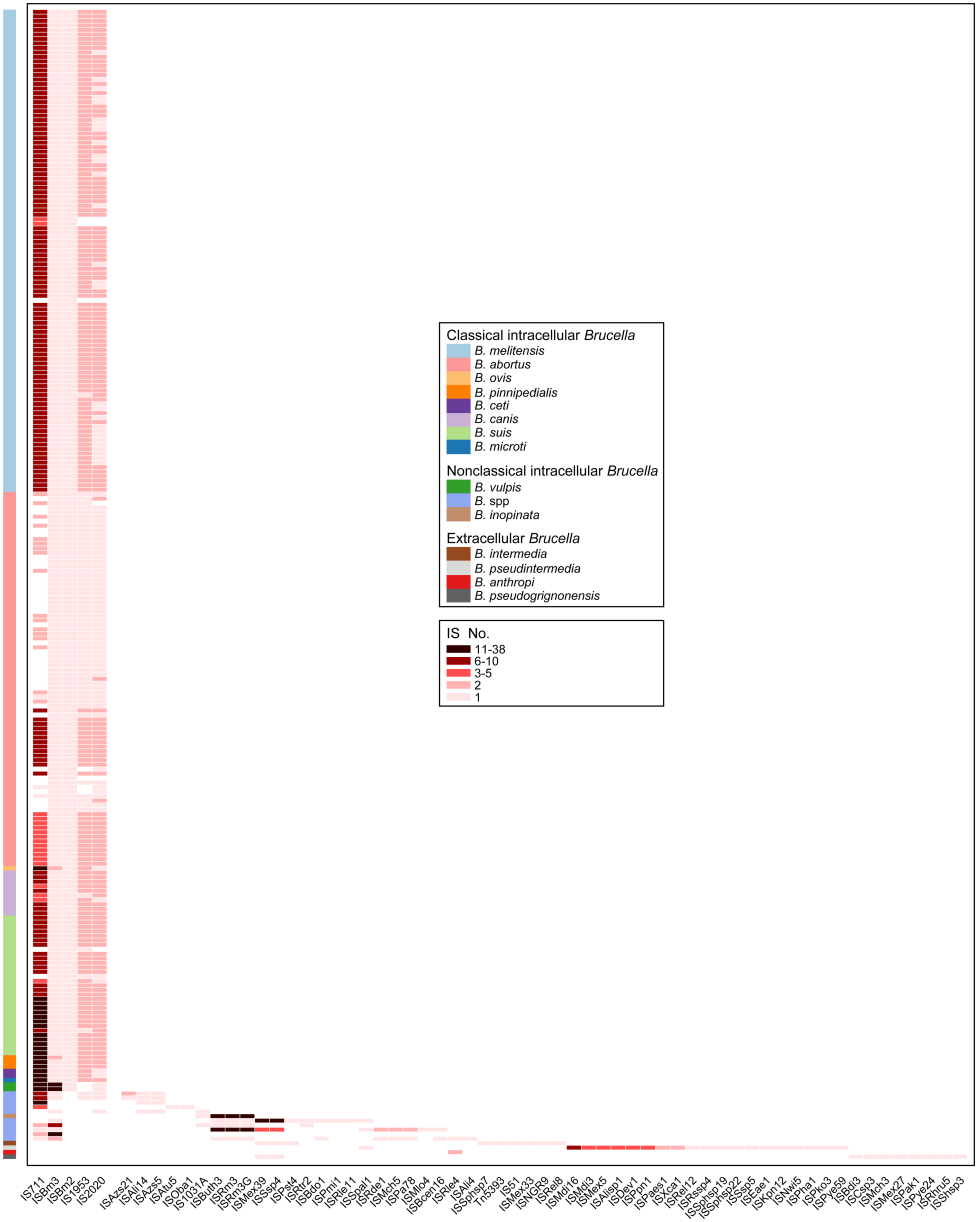
Characteristics of the types and copy numbers of IS elements carried by each genome of different *Brucella* species.

The types of all IS elements present in the 255 *Brucella* genomes were further analyzed. A total of 62 types of IS elements were identified (Figure 5). Intact copies of IS711, ISBm3, ISBm2, IS1953, and IS2020 were present in almost all the genomes of classical intracellular *Brucella* without any other types of IS elements (Figure 5). ISBm3 and ISBm2 were mostly present as single copies in these genomes. IS1953 and IS2020 were mostly present as double copies in these genomes. The number of IS711 was significantly greater than that of the other four IS elements, with an average of 8 copies per genome. Different species of classical intracellular *Brucella* possessed unique copy numbers of IS711, and Chr I of these *Brucella* strains contained more IS711 than Chr II (Supplementary Table S4). *B. ovis* contained the highest number of IS711, with 26 IS711 present on Chr I and 12 on Chr II. *B. pinnipedialis* contained the second highest number of IS711, with 21 IS711 present on Chr I and 10 on Chr II. The number of IS711 present in the genomes of other species of classical intracellular *Brucella* did not exceed 15. Furthermore, the aforementioned five types of IS elements were also present in some genomes of nonclassical intracellular *Brucella* species, but none of these genomes simultaneously harbored all five types of IS elements. Apart from these five types of IS elements with intact copies, 24 other types of truncated IS elements were distributed in the genomes of nonclassical intracellular *Brucella* species, with completeness less than 6%. Moreover, none of the genomes of extracellular *Brucella* contained the five aforementioned types of IS elements. *B. anthropi* did not possess any unique IS elements. The other three extracellular *Brucella* species, namely, *B. intermedia*, *B. pseudintermedia*, and *B. pseudogrignonensis*, harbored 6, 19, and 18 unique IS elements, respectively. Among them, ISKpn12, ISPaes1, and ISPye59 were unique to *B. pseudintermedia* and exhibited completeness levels of 100, 100, and 31.28%, respectively. The completeness of the other IS elements were less than 6%. In summary, the genomes of classical intracellular *Brucella* strains almost universally contained intact copies of IS711, ISBm3, ISBm2, IS1953, and IS2020. The genomes of nonclassical intracellular *Brucella* and extracellular *Brucella* contained residual components of many other types of IS elements, which are completely absent in classical intracellular *Brucella* species.

The residual IS components act as “scars” in the genome and represent traces of numerous ancestral transposition events; these components can provide important insights into the evolutionary history of host genomes (Siguier et al., 2014). The ancient ancestors of the *Brucella* genus are typically considered a group of free-living bacteria distributed in environments such as soil (Suarez-Esquivel et al., 2020). As the ancestors of the *Brucella* genus evolved from extracellular to intracellular bacteria, many non-essential genes on their genomes were eliminated through pseudogenization, leading to continuous genome reduction (Liu et al., 2004). IS elements participated in the pseudogenization process by transposition, and they were partially or completely lost as the genome shrank (Siguier et al., 2014). This study found that extracellular *Brucella* and nonclassical intracellular *Brucella* contained many residual traces of IS elements, while classical intracellular *Brucella* completely lacked these residual traces. This may indicate, to better adapt to their respective environments, that classical intracellular *Brucella* underwent higher levels of pseudogenization and genome reduction compared to nonclassical intracellular *Brucella* and extracellular *Brucella*.

The number of IS711 among the same *Brucella* species was comparatively conserved (Ouahrani et al., 1993). Due to its species-specific nature, IS711 has been utilized for the identification of different *Brucella* species (Paul et al., 2020). However, in our study, some genomes of *B. melitensis*, *B. abortus*, *B. suis*, and undefined *Brucella* strains did not contain IS711. Thus, there are limitations to using IS711 for identifying *Brucella* species, and caution should be exercised when applying this method in practical use. Furthermore, Ocampo-Sosa et al. reported that under laboratory conditions, IS711 in the genomes of *B. ovis* and *B. pinnipedialis* exhibited transpositional activity, while no similar phenomenon was detected for *B. melitensis* or *B. abortus* (Ocampo-Sosa and Garcia-Lobo, 2008). *B. ovis* and *B. pinnipedialis* possessed a significantly high number of IS711 copies, with 38 and 37 copies, respectively. In our study, *B. vulpis* also had a high number (27) of IS711 copies. Determining whether IS711 in *B. vulpis* could exhibit transpositional activity under laboratory conditions, similar to that of *B. ovis* and *B. pinnipedialis*, requires further exploration.

#### 3.4.2 Prophages

Prophages, which are a type of MGE, can integrate into the bacterial genome, influencing genetic variation, biological characteristics, and the epidemiology of bacteria (Canchaya et al., 2003; Kaden et al., 2014). In our study, 775 prophages (19 intact prophages, 23 questionable prophages, and 733 incomplete prophages) were identified in 255 *Brucella* genomes, with an average of 3 prophages per genome (Figure 4, Supplementary Table S5). Except for a few intact prophages, the vast majority of prophages in the *Brucella* genomes were defective. These defective prophages may represent ancient components of *Brucella* genomes (Canchaya et al., 2003). Among the 255 *Brucella* genomes, 147 genomes harbored prophages exclusively on Chr I, including 106 *B. melitensis* genomes, 15 *B. abortus* genomes, 14 *B. suis* genomes, 8 *B. canis* genomes, 1 *B. ceti* genome, 2 *B. pinnipedialis* genomes, and 1 *B. microti* genome. The remaining 108 genomes harbored prophages on both Chr I and Chr II. Moreover, except for the genomes of *B. pseudintermedia* ASAG_D25 and *B. ceti* TE28753-12, Chr I of the remaining 253 genomes harbored more prophages than Chr II, with an average of 3 and 1 prophages, respectively. Chr I and Chr II of *B. pseudintermedia* ASAG_D25 harbored 2 and 8 prophages, respectively. Chr I and Chr II of *B. ceti* TE28753-12 harbored 2 and 3 prophages, respectively. Furthermore, the average length of the prophages on Chr I was significantly greater than that on Chr II, specifically 17.3 Kb and 8.8 Kb, respectively. In addition, different *Brucella* species harbored varying numbers of prophages. The genome of *B. sp.* 2280 had the highest number of prophages, with up to 12. The genome of *B. pseudintermedia* ASAG_D25 contained the second highest number of prophages, with up to 10. The genomes of *B. vulpis*, *B. inopinata*, *B. intermedia*, and other undefined *Brucella* strains had an average of 6 prophages. The remaining *Brucella* genomes had an average of 3 prophages.

Prophages play significant roles in bacterial evolution and can facilitate the genetic diversity of bacteria (Canchaya et al., 2003). Studies have shown that most prophages in bacterial genomes are defective and in a state of gradual decay (Canchaya et al., 2003). Furthermore, research has found that *Brucella*’s Chr II contains more pseudogenes than Chr I, indicating a higher degree of genome reduction on Chr II (Zhong et al., 2012). In our study, the majority of prophages in the *Brucella* genus were found to be incomplete, the number of prophages on Chr II was fewer and their average length was lower compared to Chr I, suggesting that as the two chromosomes of *Brucella* underwent different degrees of decay, the prophage components gradually diminished, with more severe decay on Chr II. In addition, there were significant differences in the content of prophages among different *Brucella* species, especially with genomes of nonclassical intracellular *Brucella* containing a greater number of prophages, indicating a significant relationship between prophages and the genetic diversity of *Brucella*.

**Figure 6:**
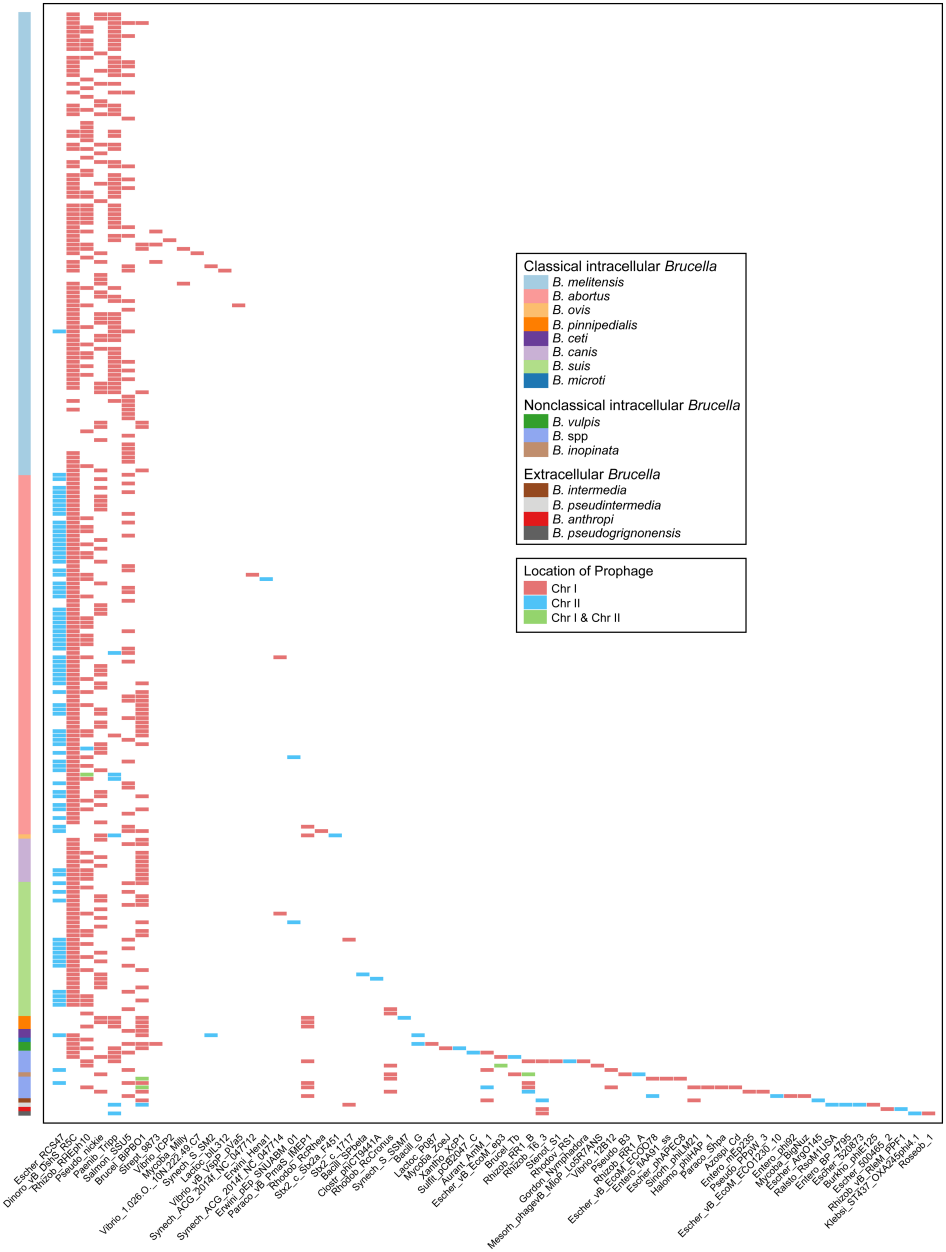
Characteristics of the types and chromosomal locations of prophages carried by the genomes of different *Brucella* species.

The types of prophages in the *Brucella* genomes were further analyzed. The 775 prophages were classified into 64 different types according to the bacteriophage genes carried by the prophages (Figure 6). Among these types, 35 were exclusively distributed on Chr I of *Brucella*, 19 were exclusively distributed on Chr II, and the remaining 10 were distributed on both Chr I and Chr II. Escher_RCS47, Dinoro_vB_DshS_R5C, Rhizob_RHEph10, Pseudo_nickie, Paenib_Tripp, Salmon_SSU5, and Brucel_BiPBO1 were relatively widely distributed in the *Brucella* genus. Brucel_BiPBO1 had the widest distribution range and was found in at least 10 species, including *B. melitensis*, *B. abortus*, *B. suis*, *B. canis*, *B. ceti*, *B. pinnipedialis*, *B. vulpis*, *B. intermedia*, *B. pseudintermedia*, and some undefined *Brucella* strains. Escher_RCS47 was exclusively distributed on Chr II of *Brucella*, while the remaining 6 types were primarily distributed on Chr I. Moreover, the other 57 types of prophages were distributed in specific *Brucella* species.

Several studies on prophages in the *Brucella* genus have been published to date. Studies have shown that prophages are related to the genetic diversity of *B. canis* (Kaden et al., 2014) and *B. melitensis* (Azam et al., 2016; Abou Zaki et al., 2017). Active phages were successfully induced by Hammerl et al. from intracellular *Brucella* (Hammerl et al., 2016) and by Jackel et al. from extracellular *Brucella* (Jackel et al., 2017). In our study, a more comprehensive investigation of prophages of the entire *Brucella* genus was conducted. The aforementioned five studies employed PHAST (Zhou et al., 2011) for the prediction and analysis of prophages. However, PHASTER was utilized in our study. PHASTER exhibits higher accuracy and sensitivity than PHAST (Arndt et al., 2016). Some *Brucella* strains from the aforementioned five studies were included, and additional prophages were indeed identified in these strains in our study. For example, Kaden et al. identified an intact Roseobacter prophage (72.5 Kb) on Chr I of the *B. canis* strain SVA13 (Kaden et al., 2014). In our study, two additional incomplete prophages were found in other regions on Chr I of *B. canis* strain SVA13, with sizes of 41.2 Kb and 19.5 Kb, respectively. Similar results were found in other strains, including *B. melitensis* M28 (Azam et al., 2016), *B. abortus* S19, *B. suis* 1330, *B. sp.* BO2 (Hammerl et al., 2016), and *B. pseudogrignonensis* K8 (Jackel et al., 2017). These new findings further revealed the importance of prophages in enhancing the genetic diversity of the *Brucella* genus. BiPBO1 (46,877 bp) was the first temperate phage induced from the *Brucella* genus (Hammerl et al., 2016). In our study, the prophage Brucel_BiPBO1 was widely distributed among various species of intracellular *Brucella* and extracellular *Brucella*, the length of which ranged from 7.2 Kb to 41.9 Kb. Some of these Brucel_BiPBO1 prophages may be inducible and deserve further exploration. In addition, some intact prophages were identified in *B. abortus* BDW, *B. suis* ZW043, and seven undefined *Brucella* strains in our study. These intact prophages may also be inducible and deserve further exploration.

#### 3.4.3 ICEs and IMEs

Integrative conjugative elements (ICEs) are a type of mobile genetic elements (MGEs) that can be excised, transferred, and integrated into the host genome (Siguier et al., 2014). ICEs are composed of three modules: the integration/excision module, the conjugation module, and the adaptation modules (Partridge et al., 2018). In our study, a total of 43 putative ICEs were identified in 42 *Brucella* genomes, including 27 *B. suis* genomes, 10 *B. canis* genomes, 1 *B. microti* genome, 2 *B. vulpis* genomes, 1 *B. pseudintermedia* genome, and 1 *B. pseudogrignonensis* genome (Figure 4; Supplementary Table S6). On Chr I of *B. pseudogrignonensis* K8, an ICE with a length of 84,516 bp was identified, representing 1.69% of the entire genome (Supplementary Table S6). The remaining 42 ICEs distributed on Chr II ranged in length from 169,588 bp to 225,620 bp and accounted for 4.19–5.83% of the entire genome (Supplementary Table S6). Moreover, except for the ICE in the genome of *B. suis* 2011017258, the GC content of the other 42 ICEs was significantly different from the GC content of their respective genomes, suggesting that these ICEs might have been acquired through HGT. Furthermore, Lavigne et al. found that the IncP island, which carries the CP4-like integrase gene *bciA* that encodes a functional integrase, was present in the genomes of *Brucella suis* biovars 1 to 4, *B. canis*, *B. neotomae*, and strains isolated from marine mammals but not in *B. melitensis*, *B. abortus*, *B. ovis*, or *B. suis* biovar 5 (Lavigne et al., 2005). In our study, all the ICEs of *B. suis*, *B. canis*, and *B. microti* harbored a complete IncP island, which accounted for 10% of the entire ICE (Supplementary Table S6). The IncP island may play a crucial role in the horizontal transfer of these ICEs in *B. suis*, *B. canis*, and *B. microti*.

Integrative mobilizable elements (IMEs) are a type of MGE that harbors the genes responsible for excision and integration, without the inclusion of any genes associated with conjugative transfer (Bellanger et al., 2014). In our study, a total of 12 putative IMEs were identified in the genomes of 2 *B. vulpis* strains, 1 *B. pseudogrignonensis* strain, and 6 undefined *Brucella* strains (Figure 4; Supplementary Table S7). The genomes of *B. pseudogrignonensis* K8, *B. sp.* 09RB8471, and *B. sp.* 10RB9215 all contained 2 putative IMEs, while the remaining 6 genomes all contained 1 putative IME. The GC content of these IMEs varied from 51.28 to 62.34% (Supplementary Table S7). The lengths of these IMEs were significantly shorter than those of the ICEs in *Brucella* genomes, ranging from 2,621 bp to 51,821 bp and accounting for 0.08–1.51% of the genome (Supplementary Table S7).

In addition to *B. suis* and *B. canis* being the classical intracellular *Brucella*, the ICEs and IMEs identified in our study were found predominantly in nonclassical intracellular *Brucella* and extracellular *Brucella*. Only the genomes of *B. vulpis* and *B. pseudogrignonensis* contained both ICEs and IMEs. These findings collectively revealed that ICEs and IMEs mainly contributed to the genetic diversity of nonclassical intracellular *Brucella* and extracellular *Brucella*.

### 3.5 The classical intracellular *Brucella*, nonclassical intracellular *Brucella*, and extracellular *Brucella* exhibited significantly different pathogenic potentials

**Figure 7:**
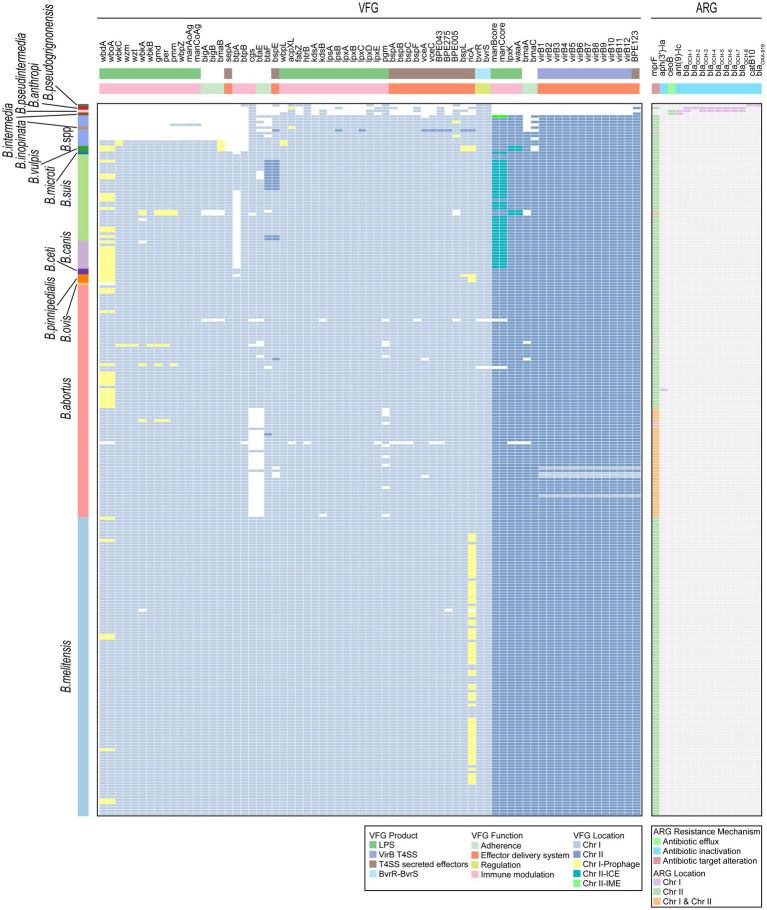
Distribution of VFGs and ARGs in the *Brucella* genus. Two heatmaps individually show the presence of VFGs and ARGs carried by each *Brucella* genome. The squares in the heatmaps are colored differently to indicate whether the corresponding VFG or ARG was distributed on the MGEs of Chr I or Chr II. The VFG product information, the resistance mechanisms of ARGs, and the corresponding species of each *Brucella* strain are also shown.

Virulence factors are properties (i.e., gene products) that enable a microorganism to establish itself on or within a host of a particular species and enhance its potential to cause disease (Liu et al., 2022). In our study, a total of 69 VFGs were identified in 255 *Brucella* genomes (Figure 7; Supplementary Table S8). Among these VFGs, 50 were widely distributed on Chr I of *Brucella*, while the remaining 19 were primarily distributed on Chr II of *Brucella*. Several VFGs, including *acpXL*, *bmaB*/*omaA*, and others, were distributed on MGEs in some *Brucella* genomes. Specifically, *wbdA* and *wboA* in *B. abortus*, *B. suis*, *B. canis*, *B. ceti*, *B. pinnipedialis*, and *B. vulpis*, as well as *ricA* in *B. melitensis*, *B. pinnipedialis*, and *B. vulpis*, were widely distributed on prophages of Chr I. Furthermore, *manBcore* and *manCcore* in *B. suis*, *B. canis*, and *B. microti* were mainly distributed on the ICEs of Chr II. The *lpxK* and *waaA* genes of *B. vulpis* were distributed on the IMEs of Chr II.

The classical intracellular *Brucella* possessed a relatively rich repertoire of VFGs, with each species containing 67–69 different VFGs (Figure 7). *B. ovis* lacked *wbdA* and *wboA*. *B. canis* did not possess *btpA*. Among the strains of nonclassical intracellular *Brucella*, *B. vulpis* strains, *B. sp.* 1315, and *B. sp.* 2594 possessed a considerable number of VFGs, with each strain harboring 65–68 different VFGs (Figure 7). Other nonclassical intracellular *Brucella* strains possessed fewer VFGs, with each strain containing 46–50 VFGs (Figure 7). In comparison to classical intracellular *Brucella* strains, these nonclassical intracellular *Brucella* strains lacked some VFGs associated with adherence and immune modulation (LPS) (Figure 7). The extracellular *Brucella* strains, which included *B. intermedia*, *B. pseudintermedia*, *B. anthropi*, and *B. pseudogrignonensis*, harbored the fewest VFGs, with each species possessing only 11–13 VFGs (Figure 7). These VFGs were predominantly located on Chr I of extracellular *Brucella*, with very few VFGs located on Chr II.

Intracellular *Brucella* is an important human and veterinary pathogen that is capable of causing brucellosis (Roop et al., 2021). In contrast, extracellular *Brucella* species represent a diverse group of free-living *Brucella*, among which only a few species are opportunistic pathogens that occasionally infect humans (Leclercq et al., 2019). In our study, intracellular *Brucella* possessed significantly more VFGs than did the extracellular *Brucella*. These VFGs were mainly involved in adherence, host immune modulation, and effector delivery systems and may be key factors leading to the difference in the pathogenicity of intracellular and extracellular *Brucella*. Additionally, some strains of nonclassical intracellular *Brucella* lacked a considerable number of VFGs associated with adherence and host immune modulation. These VFGs may cause differences in the pathogenicity between nonclassical intracellular *Brucella* strains and classical intracellular *Brucella* strains. Furthermore, although the distribution of VFGs is relatively conserved in classical intracellular *Brucella* species, *B. ovis* lacked *wbdA* and *wboA*, and *B. canis* lacked *btpA* in this study. Both *wbdA* and *wboA* are related to the biosynthesis of the lipopolysaccharide O-chain (Prasolova et al., 2023). *btpA* is related to the regulation of the host immune response (Salcedo et al., 2013). Vizcaino et al. reported the absence of *wboA* in *B. ovis* (Vizcaino et al., 2004). To our knowledge, there have been no other reports on the absence of *wbdA* in *B. ovis* or the absence of *btpA* in *B. canis*, and our research is the first to identify this phenomenon. In summary, different *Brucella* species exhibit diverse distribution patterns of VFGs, and these polymorphisms are likely closely related to variations in virulence, pathogenicity, and lifestyle among different *Brucella* species.

### 3.6 Intracellular and extracellular *Brucella* strains showed differences in genotypic resistance profile

The inappropriate use of antibiotics has promoted a widespread increase in antibiotic resistance in bacteria. The HGT of ARGs encoded on MGEs is one of the key driving factors in this process (Botelho and Schulenburg, 2021). In our study, a total of 14 ARGs were identified in the 255 *Brucella* genomes (Figure 7; Supplementary Table S9). These ARGs confer antibiotic resistance through mechanisms such as antibiotic efflux, antibiotic inactivation, and antibiotic target alteration. *mprF* confers resistance to peptide antibiotics. *Ant(9)-Ic* and *aph(3′)-Ia* confer resistance to aminoglycoside antibiotics. *catB10* confers resistance to phenicol antibiotics. It should be noted that *catB10* only exhibits a query coverage of 73% in this study, and this incomplete sequence of *catB10* may result in non-functional resistance of *Brucella*. *ceoB* confers resistance to aminoglycoside and fluoroquinolone antibiotics. *bla*_OXA-919_ confers resistance to penam antibiotics. The remaining 8 ARGs confer resistance to penem, penam, cephamycin, cephalosporin, and monobactam antibiotics. There are nine ARGs that are beta-lactamase genes, including *bla*_OCH-1_, *bla*_OCH-2_, *bla*_OCH-3_, *bla*_OCH-4_, *bla*_OCH-5_, *bla*_OCH-6_, *bla*_OCH-7_, *bla*_OCH-8_, and *bla*_OXA-919_. Moreover, *mprF* and *ceoB* were located primarily on Chr II of *Brucella*, while the remaining 12 ARGs were distributed only on Chr I (Figure 7). Furthermore, except for *aph(3′)-Ia* and *mprF*, the remaining 12 ARGs were distributed only among extracellular *Brucella*. The genome of *B. abortus* clpP contained both *aph(3′)-Ia* and *mprF*. The other intracellular *Brucella* strains contained only *mprF*.

In this study, various MGEs in *Brucella* genomes, including prophages, ICEs, and IMEs, were devoid of any ARGs, suggesting that the role of MGEs in the dissemination of ARGs might be limited in the *Brucella* genus. Furthermore, all the intracellular *Brucella* strains commonly harbored only one ARG, *mprF*. The remaining 12 ARGs were exclusively present in extracellular *Brucella*, suggesting that these two types of *Brucella* may have significantly different antibiotic resistance. Intracellular *Brucella* and extracellular *Brucella* exhibit distinct survival habits. The parasitism of intracellular *Brucella* may have limited their acquisition of ARGs through HGT. In contrast, extracellular *Brucella* species exist mainly in extracellular environments, which facilitates frequent genetic exchange and the acquisition of ARGs. In addition, recent studies have revealed that the resistance of *Brucella* spp. to various antibiotics, such as rifampicin, azithromycin, tetracycline, and so on, has been increasing (Wareth et al., 2021, 2022; Celik et al., 2023; Rezaei Shahrabi et al., 2023; Beig et al., 2024). These studies indicate an increase in antibiotic resistance in intracellular *Brucella*, which urgently requires attention. However, the distribution of ARGs within the *Brucella* genus was found to be very limited in our study. This may be attributed to the limitations of the CARD database that was used in our research, which may lack sufficient information on *Brucella* ARGs. In addition, more complex mechanisms may also contribute to drug resistance in *Brucella*.

## 4 Conclusion

In this study, a comparative genomic analysis of the *Brucella* genus was conducted using publicly available genomic data. The *Brucella* genus was found to possess a dynamic and open pangenome, with Chr II exhibiting a higher level of dynamism, openness, and plasticity than Chr I. On Chr I of *Brucella*, the core gene families were predominant, while on Chr II, the noncore gene families were predominant. Moreover, *Brucella* genomes were found to harbor some gene families acquired through HGT; these gene families were distributed mainly on Chr II and consisted primarily of noncore gene families. Furthermore, various MGEs, such as IS elements, prophages, ICEs, and IMEs, exhibited significantly different distribution patterns between Chr I and Chr II of *Brucella*. Additionally, different species of classical intracellular *Brucella*, nonclassical intracellular *Brucella*, and extracellular *Brucella* exhibited different levels of HGT and different distribution patterns of MGEs, VFGs, and ARGs. These differences among different *Brucella* species may be of significant importance for the genetic diversity of the *Brucella* genus, and to some extent could explain the diverse phenotypic characteristics exhibited by *Brucella* species. Due to limitations of the currently available public genomic data, the dataset used in this study could not encompass all species within the *Brucella* genus. In particular, the limited number of available genomes from extracellular *Brucella* and several other species in this study compromised the generality of some results. Thus, incorporating additional *Brucella* genomes in future research is necessary to acquire a more comprehensive understanding of the genetic evolutionary characteristics of the entire *Brucella* genus.

## Data availability statement

The original contributions presented in the study are included in the Supplementary material, further inquiries can be directed to the corresponding authors.

## Author contributions

ZY: Conceptualization, Methodology, Software, Visualization, Writing – original draft. ZC: Conceptualization, Methodology, Software, Visualization, Writing – review & editing. XW: Investigation, Resources, Writing – review & editing. ZZ: Software, Validation, Writing – review & editing. FZ: Data curation, Project administration, Writing – review & editing. FK: Formal analysis, Validation, Writing – review & editing. WL: Software, Writing – review & editing. HR: Funding acquisition, Supervision, Writing – review & editing. YJ: Conceptualization, Funding acquisition, Supervision, Writing – review & editing. JY: Conceptualization, Funding acquisition, Supervision, Writing – review & editing.
