# Supplementary material for: Comparative genomic analysis provides insights into the genetic diversity and pathogenicity of the genus Brucella
Source: Front Microbiol. 2024 Apr 24;15:1389859. doi: 10.3389/fmicb.2024.1389859 (PMC11076708; doi:10.3389/fmicb.2024.1389859)
Supplement: Supplementary file 2 [file Data_Sheet_1.PDF]

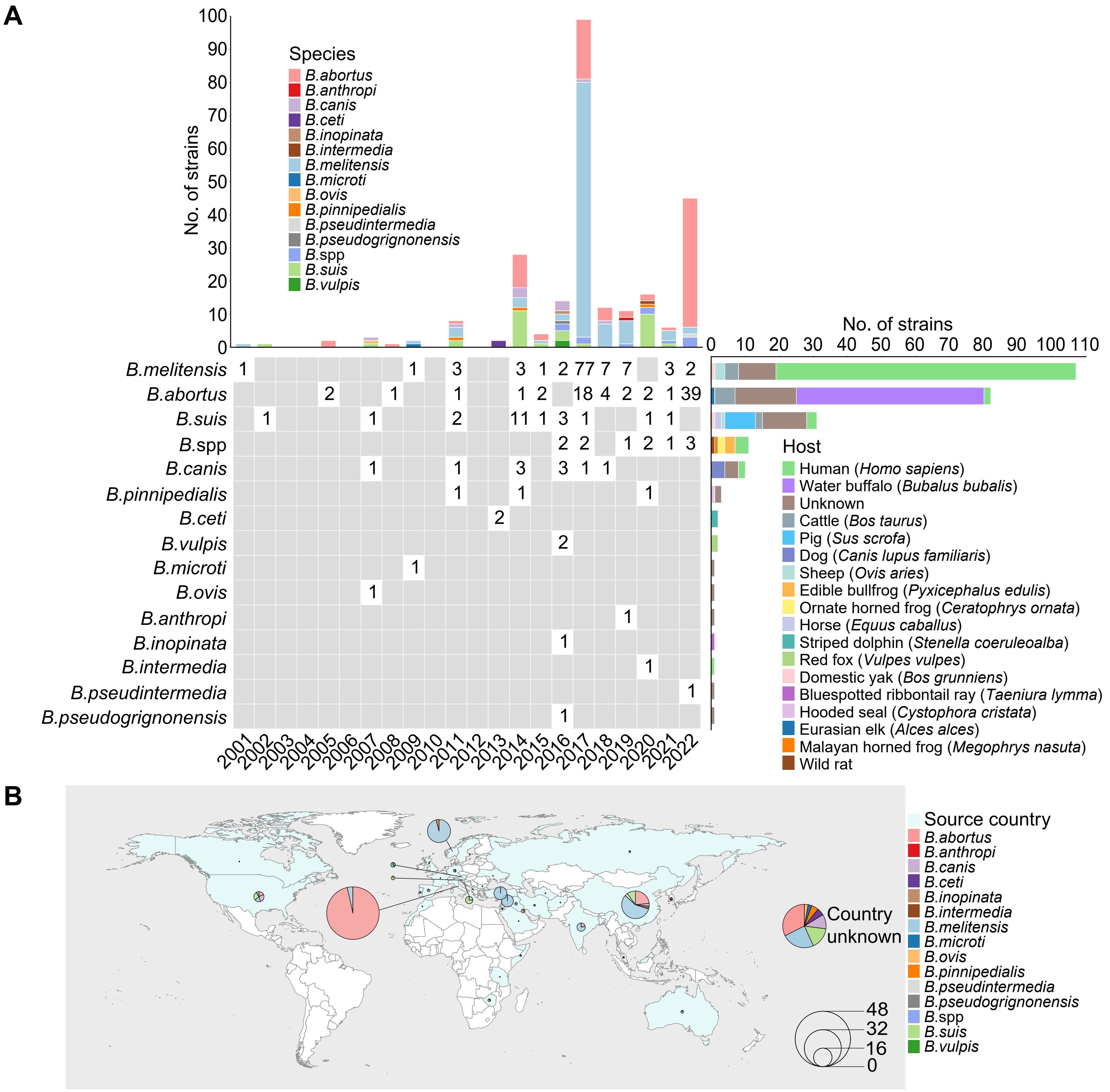

Supplementary Figure S1. Sample information of the 255 *Brucella* strains. (A) The heatmap and the bar plot above show the number of strains isolated from different years for different *Brucella* species in the dataset, with the bar plot on the right showing the host composition of all strains for different *Brucella* species. (B) The map shows the geographical distribution of all strains and the species composition in each region in the dataset.

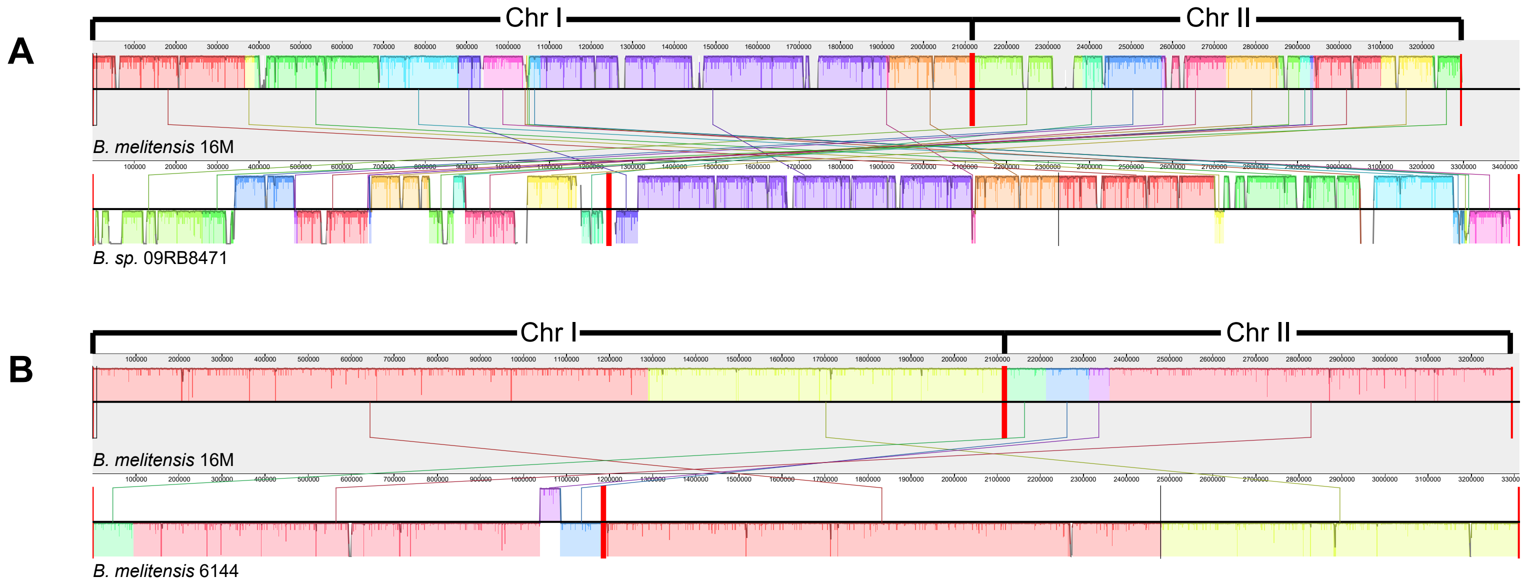

Supplementary Figure S2. The numbering errors of two chromosomes of *B. sp*. 09RB8471 and *B. melitensis* 6144. The results of the collinearity analysis for *B. sp*. 09RB8471 (A) and *B. melitensis* 6144 (B) with the reference strain *B. melitensis* 16M indicate that the numbering order of Chr I and Chr II is reversed for both *B. sp*. 09RB8471 and *B. melitensis* 6144.
In this study, we have corrected these numbering errors.

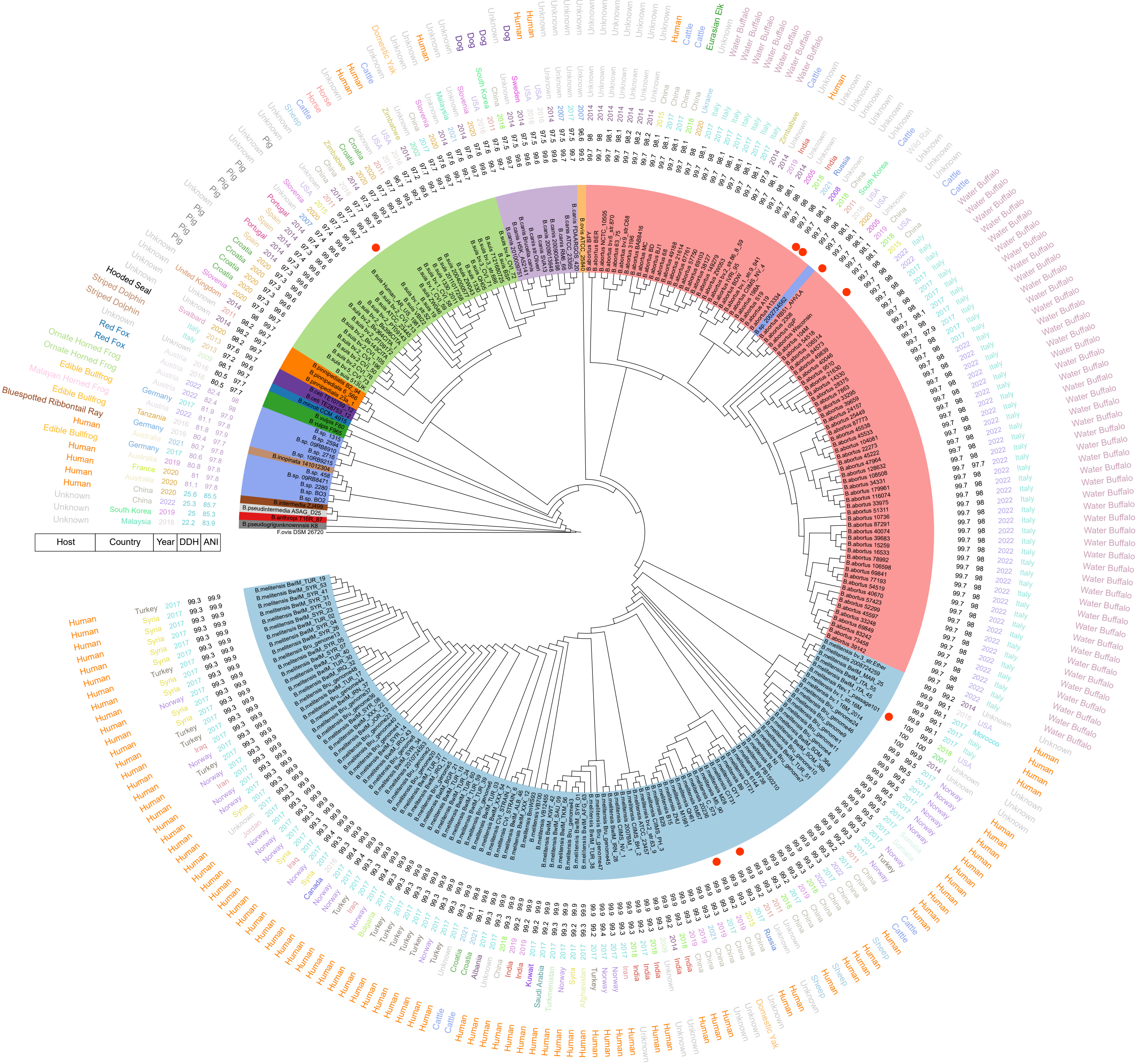

Supplementary Figure S3. Core genome phylogenomic tree of 255 *Brucella* strains.

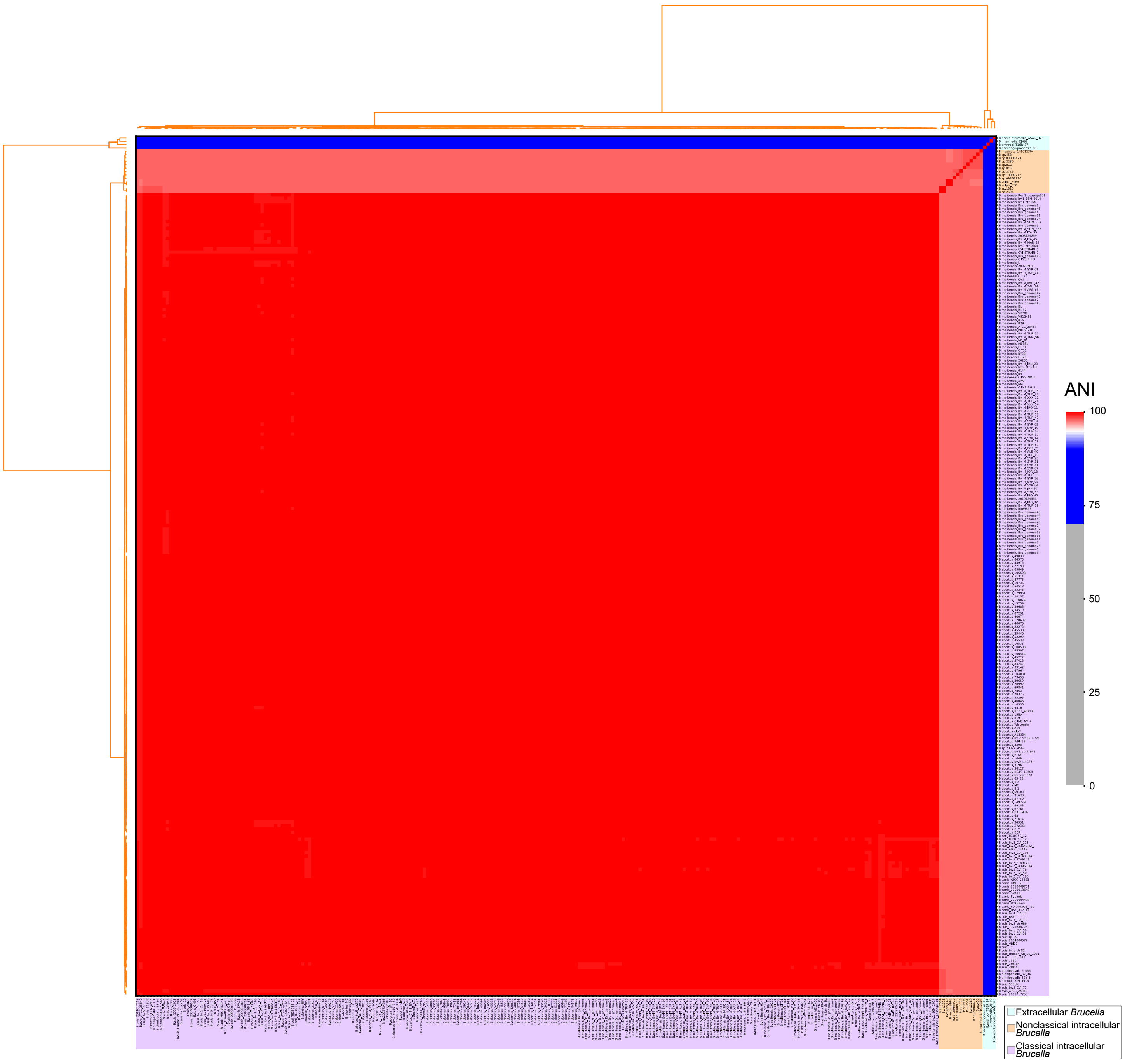

Supplementary Figure S4. The ANI heatmap of pairwise comparisons among 255 *Brucella* genomes.

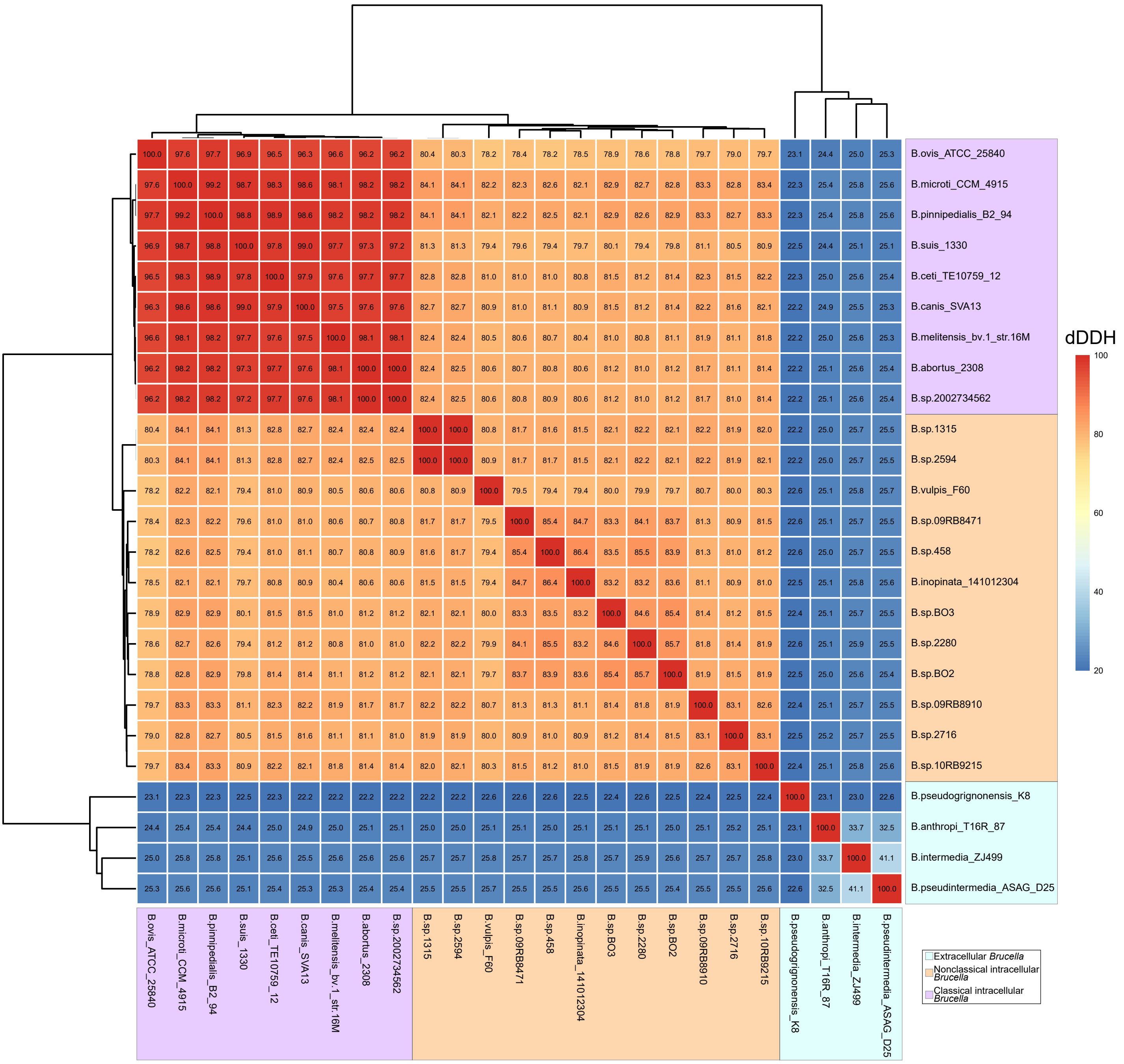

Supplementary Figure S5. The dDDH heatmap of pairwise comparisons among 25 reference genomes from different *Brucella* species.

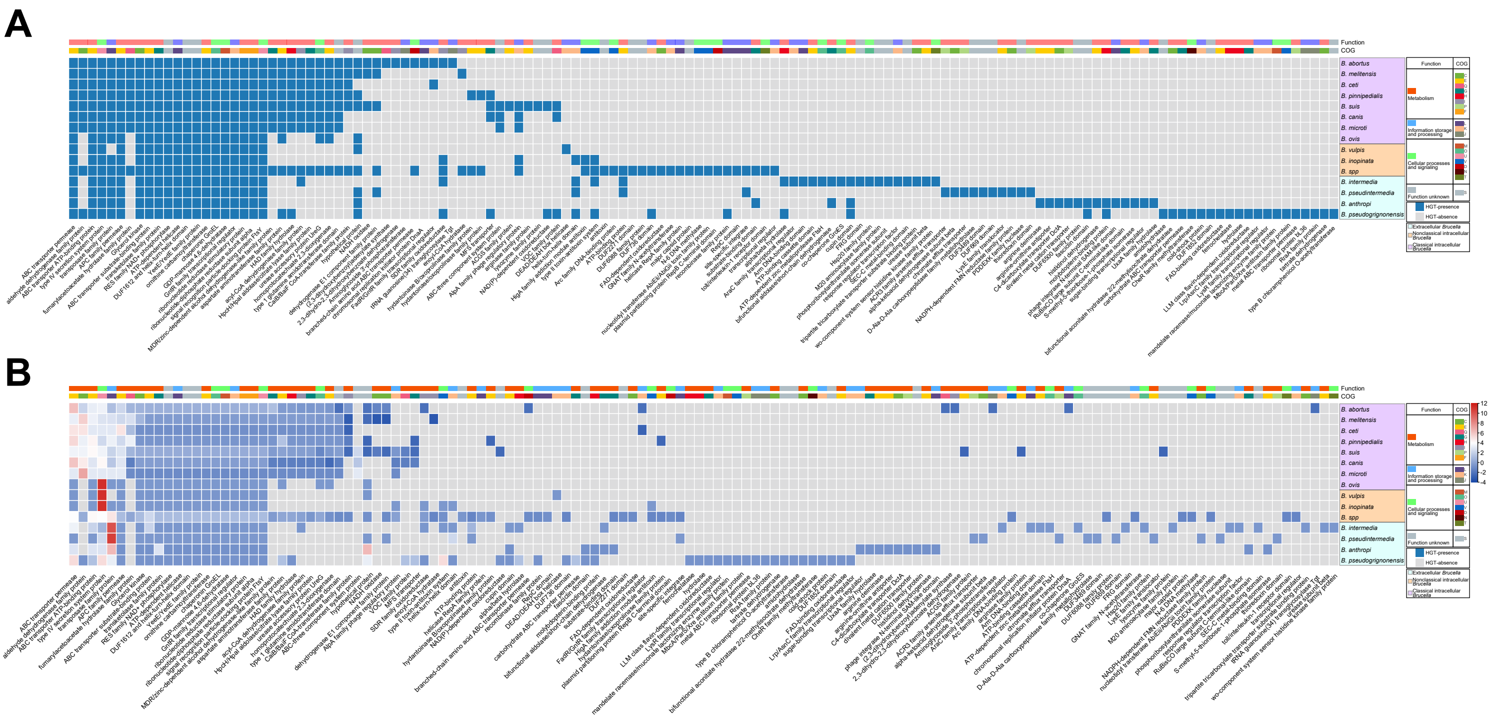

Supplementary Figure S6. The products encoded by horizontal transfer gene families (HTGFs) of different *Brucella* species. (A) The heatmap shows the presence or absence of HTGFs encoding different products in different *Brucella* species. (B) The heatmap shows the abundance of HTGFs encoding different products in different *Brucella* species.

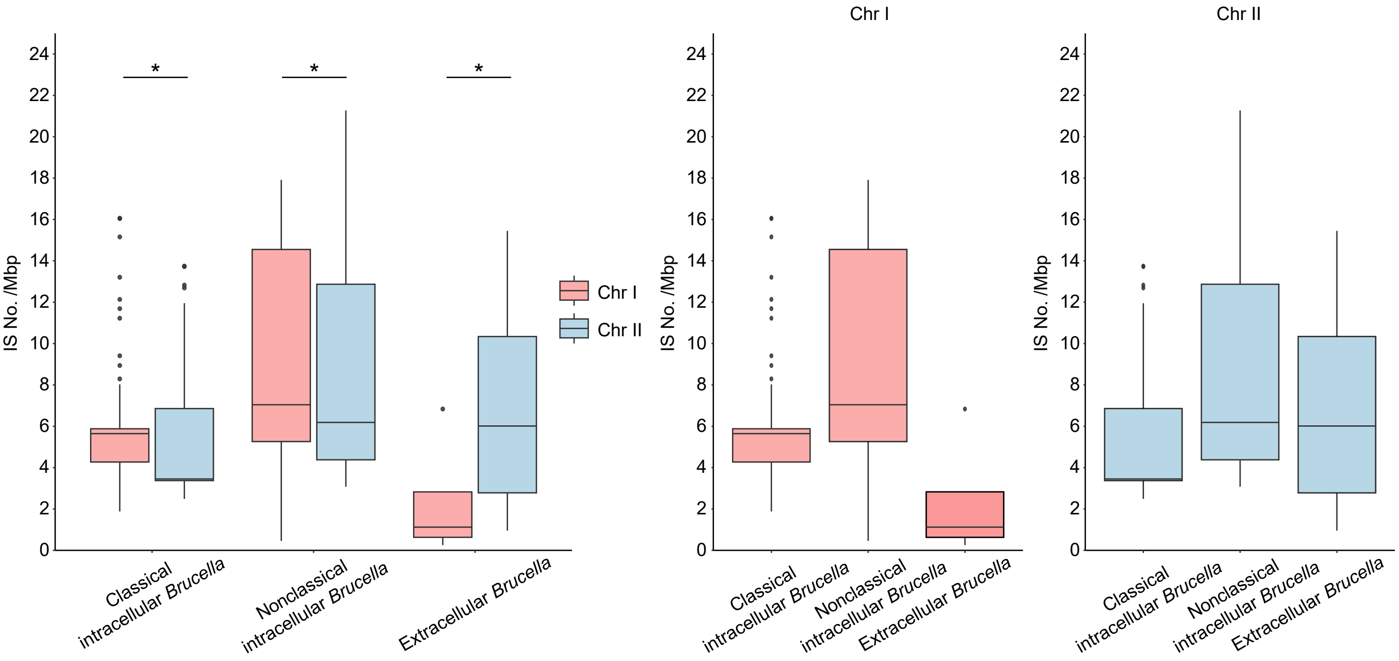

Supplementary Figure S7. The density of insertion sequences on the two chromosomes of classical intracellular *Brucella*, nonclassical intracellular *Brucella*, and extracellular *Brucella*.
